# The genus *Scaphidium* Olivier in East China (Coleoptera, Staphylinidae, Scaphidiinae)

**DOI:** 10.3897/zookeys.403.7220

**Published:** 2014-04-17

**Authors:** Liang Tang, Li-Zhen Li, Wen-Jia He

**Affiliations:** 1 Department of Biology, Shanghai Normal University, 100 Guilin Road, Building 1-323, Shanghai, 200234 P. R. China

**Keywords:** Coleoptera, Staphylinidae, Scaphidiinae, *Scaphidium*, new species, East China

## Abstract

A review of 21 species of *Scaphidium* Olivier from East China is presented, including 6 new species: *S. jinmingi* **sp. n.** (Zhejiang, Anhui, Chongqing), *S. crypticum* **sp. n.** (Zhejiang, Fujian, Jiangxi, Guangxi), *S. varifasciatum* **sp. n.** (Zhejiang, An’hui), *S. robustum* **sp. n.** (Fujian, Guizhou, Chongqing, Guangxi, Yunnan), *S. connexum* **sp. n.** (Zhejiang, Fujian, Guangxi), and *S. bayibini* **sp. n.** (An’hui). New province records for *S. comes* Löbl, *S. grande* Gestro, *S. sauteri* Miwa & Mitono, *S. formosanum* Pic, *S. carinense* Achard, *S. sinense* Pic, *S. delatouchei* Achard, *S. biwenxuani* He, Tang & Li, *S. klapperichi* Pic, *S. stigmatinotum* Löbl, *S. wuyongxiangi* He, Tang & Li, and *S. direptum* Tang & Li as well as some biological notes are reported. Habitus and diagnostic characters of all species are photographed and a key to *Scaphidium* species of East China is provided.

## Introduction

*Scaphidium* Olivier (1790) is a large genus of the subfamily Scaphidiinae, with 338 species (including two fossil species) known world-wide (Löbl 1997 and subsequent descriptions), including 51 species from China. Our ongoing study of the Chinese *Scaphidium* reveals that the fauna of East China, which covers the Shanghai municipality and the provinces Shangdong, Jiangsu, Anhui, Zhejiang, Jiangxi and Fujian, is characteristic and of special interest. The presence of *Scaphidium* in this region is still rather unclear, and the species number has rapidly increased in recent years, pointing to inadequate collecting activities in the past. Up until the present, 13 species had been known from this region and eight of them were recorded or described in our papers since 2008. Additional specimens have been accumulated by continuous field work and these collections, in particular coming from recent trips to Anhui and Fujian, led us to review the East Chinese fauna of the genus *Scaphidium*. In addition, many collecting data based on specimens coming from other regions are used for discussing intra-specific variability and provide a better understanding of species distribution.

The collecting data suggest that the most species-rich areas in East China are Tianmushan (North Zhejiang) and Wuyishan (border of Zhejiang, Fujian and Jiangxi). Most species have been found there and especially worthy to note is Wuyishan which has two endemic species, *Scaphidium fukiense* and *Scaphidium vernicatum*. The Yaoluoping Natural Reserve in Dabieshan in West Anhui is also worth special attention: two unique species, *Scaphidium spinatum* and *Scaphidium bayibini*, are known only from there while their close relatives, *Scaphidium grande* and *Scaphidium klapperichi* are widespread.

## Material and methods

Specimens examined during the preparation of this paper were mainly collected from East China and killed with ethyl acetate. For examination of male genitalia, the last two abdominal segments were detached from the body after softening specimens in hot water. The aedeagi were mounted in Euparal (Chroma Gesellschaft Schmidt, Koengen, Germany) on plastic slides. Photos of the aedeagi were taken with a Canon G7 attached to Olympus SZX 16 stereomicroscope; photos of the antennae, front legs and habitus were taken with a Canon macro photo lens MP-E 65mm attached to a Canon EOS7D camera.

The type specimens and additional material treated in this study are deposited in the following public and private collections:

CBWX Private collection of Wen-Xuan BI, Shanghai, P. R. China

CZTX Private collection of Tie-Xiong Zhao, Zhuji, Zhejaing, P. R. China

HBUM Museum of Hebei University, P. R. China (Guo-Dong Ren)

IOZ Institute of Zoology Chinese Academy of Sciences (Hong-Bin Liang)

MHNG Muséum d’histoire naturelle, Geneva, Switzerland (Ivan Löbl)

NHRS Naturhistoriska Riksmuseet, Stockholm, Sweden (Bert Gustafsson)

NMPC Narodní Muzeum, Entomologické odd., Praha, Czech Republic (Martin Fikáček)

SEM Shanghai Entomology Museum, the Chinese Academy of Science, P. R. China (X.-W. Liu)

SHNU Department of Biology, Shanghai Normal University, P. R. China (Liang Tang)

SYSU Sun Yat-Sen University, Guangzhou, P. R. China (Feng-Long Jia)

TARI Taiwan Agricultural Research Institute, Wufeng, Taichung, Taiwan (Chi-Feng Lee)

The abbreviation BL is used for the body length, measured from the anterior margin of the clypeus to the apex of the abdomen.

## Taxonomy

### Scaphidium jinmingi sp. n.

http://zoobank.org/F0FFD88F-6CB7-4E04-87FB-C49286D55880

http://species-id.net/wiki/Scaphidium_jinmingi

Figs 1 , 2 , 58–61

#### Type material.

**Holotype.** **Zhejiang:** ♂, Lin’an City, West Tianmushan, alt. 1400 m, 10.VII.2008, Y.-X. Wu & M. Jin leg. “Holotype / *Scaphidum jinmingi*/ Tang & Li” [red handwritten label] (SHNU). **Paratypes.** **Zhejiang:** 3♂♂3♀♀, same data as the holotype (1 pair in MHNG, remaining in SHNU); 2♂♂1♀, Anji County, Longwangshan, N 30°24', E 119°21', alt. 1200–1500m, 8.IV.2012, Bi, Hu & Yin leg. (1♂ in CBWX, remaining in SHNU); 2♀♀, Lin’an City, West Tianmushan, alt. 1500 m, 17–18.V.2008, W.-X. Bi leg. (SHNU); 1♂3♀♀, Anji County, Longwangshan, N 30°23', E 119°26', alt. 1450m, 14.V.2013, C.-C. Dai leg. (SHNU). **Anhui:** 3♂♂2♀♀, Yuexi County, Yaoluoping N. R., Duozhijian, 30°58'38"N, 116°6'59"E, alt. 1650m, 19.VI.2013, Dai & Peng leg. (SHNU). **Chongqing:** 1♂, Chengkou County, East Daba Shan, lower Huang’an Gou, 31°51'227"N, 109°7'174"E, alt. 2039m, 22–23.IV.2008, H. Huang & W. Xu leg. (SHNU).

#### Description.

BL: 4.1–4.8 mm.

Body black with distinct blue to violet metallic luster, labrum light brown, antennal club (Fig. 60) blackish, tarsi dark brown.

Frons coarsely and densely punctate, punctures near inner side of eyes relatively small and a little confluent.

Pronotum slightly raised above elytra. Antebasal puncture row impressed, not interrupted at middle, with punctures very coarse and more or less longitudinal; discal punctation almost evenly coarse and rather dense, consisting of deep punctures, a little coarser than those on frons, puncture intervals mostly 0.8 to 1.5 times as large as their diameters.

Elytra with sides relatively parallel. Disc slightly impressed apically, basal and sutural stria rows impressed; discal punctation similar to that of pronotum, punctures coarse on middle portion and less coarse on lateral portion and near suture; discal puncture row absent; basal stria row with punctures about as coarse as those forming pronotal antebasal row, sutural stria puncture row relatively fine.

Prohypomera slightly uneven, with fine and very sparse punctures, without microsculpture.

Mesepisterna with very fine and sparse punctures.

Abdominal tergites with relatively coarse and dense punctures. Sternite III with dense micropunctures on basal half and lateral portions, remaining sternites with dense micropunctures only on lateral parts. Reticular microsculpture appearing rarely and irregularly on sternites.

Legs relatively short, mesotibiae and metatibiae slightly curved.

Male. Metaventrite (Fig. 2) as in female, without setiferous patch. Protibiae (Fig. 61) with ventral side weakly expanded at apical 1/4 forming a tiny blunt angle. Segments 1 to 3 of protarsi widened with dense pubescence on ventral side. Median lobe of aedeagus (Fig. 58) with sclerotized internal sac (Fig. 59) consisting of two apical sclerotized rods and subapical transverse sclerite.

**Figures 1–4.**
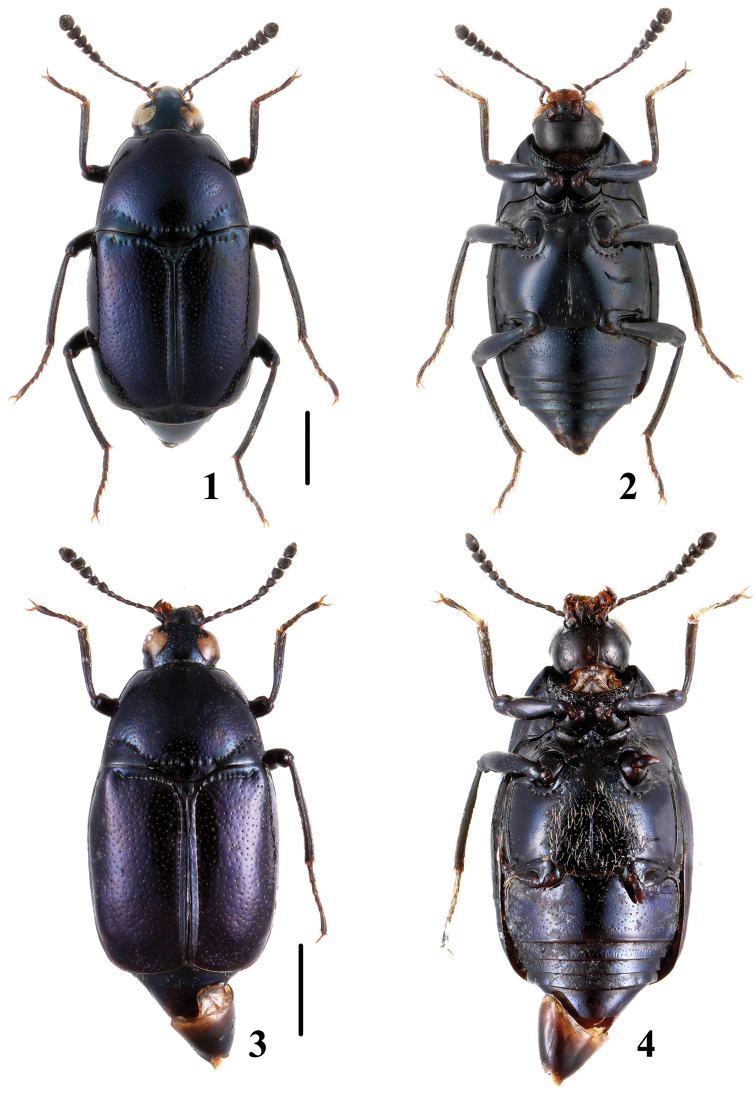
Habitus of *Scaphidium*. **1, 2** *Scaphidium jinmingi* **3, 4** *Scaphidium jizuense* (Holotype). Scales = 1 mm.

#### Distribution.

China (Zhejiang, Anhui, Chongqing).

#### Remarks.

The new species is unique within Chinese *Scaphidium* by the male metaventrite lacking a setiferous patch. The new species is very similar to *Scaphidium jizuense* Löbl, 1999 from Yunnan and *Scaphidium cyanellum* Oberthür, 1884 from Nepal and India in general appearance. It can be distinguished from *Scaphidium jizuense* by a stouter terminal antennal segment, less distinct blunt angle of the male protibiae, prohypomera without microsculpture, and with fine punctures (in *Scaphidium jizuense* prohypomera are microsculptured and without punctures), from *Scaphidium cyanellum* by the stouter terminal antennal segment and the coarser elytral punctation.

#### Etymology.

This species is named in honor of Mr. Ming Jin, who firstly collected specimens of the new species.

### Scaphidium comes

Löbl, 1968

http://species-id.net/wiki/Scaphidium_comes

Figs 5 , 6 , 62–65 , 142–144

Scaphidium comes Löbl, 1968: 388; He et al. 2008a: 181.

#### Material examined.

**Zhejiang:** 1♂5♀♀, Lin’an City, West Tianmushan, alt. 350m, 1.V.2006, Y.-X. Wu leg. (SHNU); 1♂2♀♀, same locality but alt. 300m, 29.V.2008, Huang & Yan leg. (SHNU). **Hunan:** 4♂♂6♀♀, Xiangtan City, Zhaoshan County, 30.I.2011, Z. Peng leg. (SHNU). **Hubei:** 1♂1♀, Wufeng County, Houhe N. R., 30°5'7"N, 110°33'11"E, 9.VII.2013, Dai, Peng & Xie leg. (SHNU). **Guangxi:** 6♂♂7♀♀, Shangsi County, Shiwandashan N. R., 300–400m, 23.IV.2011, Zhai, Peng & Zhu leg. (SHNU). **Hainan:** 1♂, Baishan County, Yinggeling N. R., 3.XII.2007, G.-Y. Yang leg. (SHNU)

#### Distribution.

China (Zhejiang, Hunan, Hubei, Guangxi, Hainan), North Korea.

#### Remarks.

These are new records to Hunan, Hubei, Guangxi and Hainan. The species is similar to *Scaphidium jinmingi*, but it can be easily recognized by the entirely black coloration which is metallic blue in *Scaphidium jinmingi*. The coloration of the femora is variable, being reddish in a few specimens from Zhejiang and more than half of the specimens from Hunan.

**Figures 5–8.**
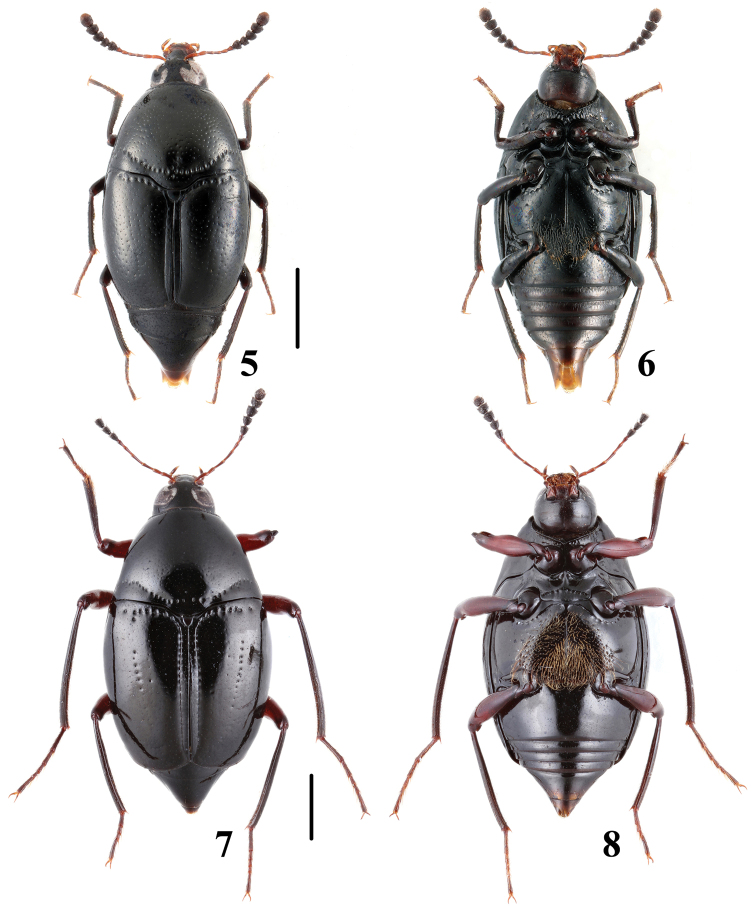
Habitus of *Scaphidium*. **5, 6** *Scaphidium comes* **7, 8** *Scaphidium fukiense*. Scales = 1 mm.

### Scaphidium fukiense

Pic, 1954

http://species-id.net/wiki/Scaphidium_fukiense

Figs 7 , 8 , 66–69

Scaphidium fukiense Pic, 1954: 58; Löbl 2009: 710.

#### Material examined.

**Lectotype.** **Fujian:** 1♂, Kuatun, 1.VIII.1946. (NHRS).

#### Other material.

**Fujian:** 1♂2♀♀, Wuyishan City, Guadun Vil., 27°44'N, 117°38'E, alt. 1200–1300m, 24.V.2012, Dai, Peng & Song leg. (SHNU).

#### Distribution.

China (Fujian).

#### Remarks.

The species is similar to *Scaphidium biseriatum* Champion, 1927 and its allies, but can be distinguished from them by the smaller body size and the reddish femora. It is possibly endemic to Wuyishan.

### Scaphidium grande

Gestro, 1879

http://species-id.net/wiki/Scaphidium_grande

Figs 9 , 10 , 70–73 , 145–150

Scaphidium grande Gestro, 1879: 50; Pic 1915: 3; Pic 1920: 189; Achard 1924: 91; Löbl 1992: 488; He et al. 2008a: 181; Tang and Li 2010a: 68.
Scaphidium grande var. *inimpressum*Pic 1920: 189; Löbl 1992: 488.
Scaphidium grande var. *subannulatum* Pic 1915: 3; Löbl 1992: 488.
Scaphidium grande var. *melanopus* Achard, 1924: 91; Löbl 1992: 488.

#### Material examined.

**Chongqing:** 1♂, Jiangjin, Simianshan, 10.VII.2005, W.-W. Zhang leg. (SHNU). **Sichuan:** 1♀, Luding, Moxi, alt. 1300m, 20.V.2009, W.-J. He leg. (SHNU). **Guizhou:** 1♀, Fanjingshan, Heiwan Reiver, 800m, 3.VIII.2001, Dong leg. (IOZ). **Hunan:** 1♀, Tongdao County, Shangyan, 24.VII.2004, J.-L. Wang (HBUM); 3♂♂2♀♀, Yanling County, Taoyuandong Park, 26°29'14"N, 114°0'42"E, alt. 770m, Dai, Peng & Xie leg. (SHNU). **Zhejiang:** 1♂1♀,Kaihua County, Gutianshan, N29°15, E118°7', alt. 770m, 16.VII.2013, Dai, Peng & Xie leg. (SHNU). **Fujian:** 1♂, Yong’an County, Xiyang, 19.IV.1962, G.-Y. JIN leg. (NO. 24110832, SEM); 1♀, Fuzhou City, Shoushan, Beifeng, V.2004, M. Li leg. (SHNU); 1♀, Wuyishan, 27.V.2002, Li Li- Zhen leg. (SHNU); 2♀♀, Wuyishan City, Guadun, 27°44'N, 117°38'E, alt. 1000–1300m, 29–30.V.2012, Song, Peng & Dai leg. (SHNU). **Guangdong:** 1♂, Nanxiong City, Yuntan street, 3.V.2007, B.-P. Huang leg. (SHNU). **Yunnan:** 1♂, Menla, alt. 670m, 21.IV.1982, Yu leg. (SHNU); 1♂, Jinghong City, 11.VI.1973, G.-T. Jin leg. (NO. 24038143, SEM); 1♂, Jinghong City, Nabanhe N. R., Manfei, alt. 630 m, 29.VII.2005, LI & LI leg. (SHNU); 1♂1♀, Menlun, Xipian, alt. 985m, 1.IV.2009, W.-X. Bi leg. (SHNU); 1♂1♀, Menla, Wangtianshu, alt. 600m, 5.IV.2009, W.-X. Bi leg. (SHNU); 1♀, Menla, Nanman Vil., alt. 900m, 7.IV.2009, W.-X. Bi leg. (SHNU); 2♂♂, Xishuangbanna botanical garden, 20.IV.2010, X.-Y. Zhu leg. (SHNU). **Guangxi:** 1♂, Rong’an County, Xishan Forest Farm, Hongchagou, 26.VII.2006, Yang leg. (SHNU); 1♀, Shangsi County, Pinglongshan, 6.IV.2002, alt. 350–500m, A.-M. Shi leg. (HBUM); 7♂♂9♀♀, Shangsi County, Shiwandashan, alt 300–500m, 23–25.IV.2011, Zhai, Peng & Zhu leg. (SHNU); 13♂♂7♀♀, Xing’an County, Mao’ershan, Gaozhai, alt. 440m, 7.8.VII.2011, He, Tang, Peng, Ma, Chen & Zhu leg. (SHNU); 2♂♂4♀♀, Jinxiu County, Yinshan Bohuzhan, alt. 1200m, 22–27.VII.2011, Peng, Hu & Yin leg. (SHNU); 2♀♀, Jinxiu County, Shengtangshan, alt. 700–900m, 28–29.VII.2011, Z. Peng leg. (SHNU) **Hainan:** 1♀, Lingshui County, Diaoluoshan, 1100m, 29.III.1999, W.-Y. Zhou leg. (SHNU); 1♂, Wuzhishan City, Shuiman, 23–25. May.2007, Ba & Lang leg. (HBUM); 1♂, Limushan, alt. 800m, 20.IV.2009, X.-Y. Zhu leg. (SHNU); 6♂♂3♀♀, Linshui County, Diaoluoshan, alt. 1000m, 18–23.IV.2010, Z.-W. Yin leg. (SHNU); 2♂♂2♀♀, Changjiang County, Bawangling, alt. 1000m, 10.IV.2010, B.-P. Huang leg. (SHNU); 2♂♂3, Ledong County, Jianfengling, alt. 1000m, 17–24.V.2011, W.-X. Bi leg. (SHNU).

#### Distribution.

China (Chongqing, Sichuan, Guizhou, Hunan, Zhejiang, Fujian, Guangdong, Yunnan, Guangxi, Hainan, Taiwan?), Nepal, Myanmar, Thailand, Laos, Malaysia, Vietnam, Indonesia.

#### Remarks.

This is new record to Zhejiang. The species is one of the most widely distributed species in China while its relatives are restricted to rather smaller areas. The main differences between these species affect the male characters (see Tang and Li 2010a). The Taiwanese records of the species are based on *Scaphidium grande* var. *inimpressum* Pic which was synonymized by Löbl in 1992. Several specimens in our collection from Taiwan are considered to be a closely related species of *Scaphidium grande*, and this might imply that *Scaphidium grande inimpressum* Pic is a good species. To clarify the doubt, a study on the type material of *Scaphidium grande inimpressum* Pic and more related material from Taiwan will be necessary; the distribution of *Scaphidium grande* in Taiwan is now doubtful.

**Figures 9–12.**
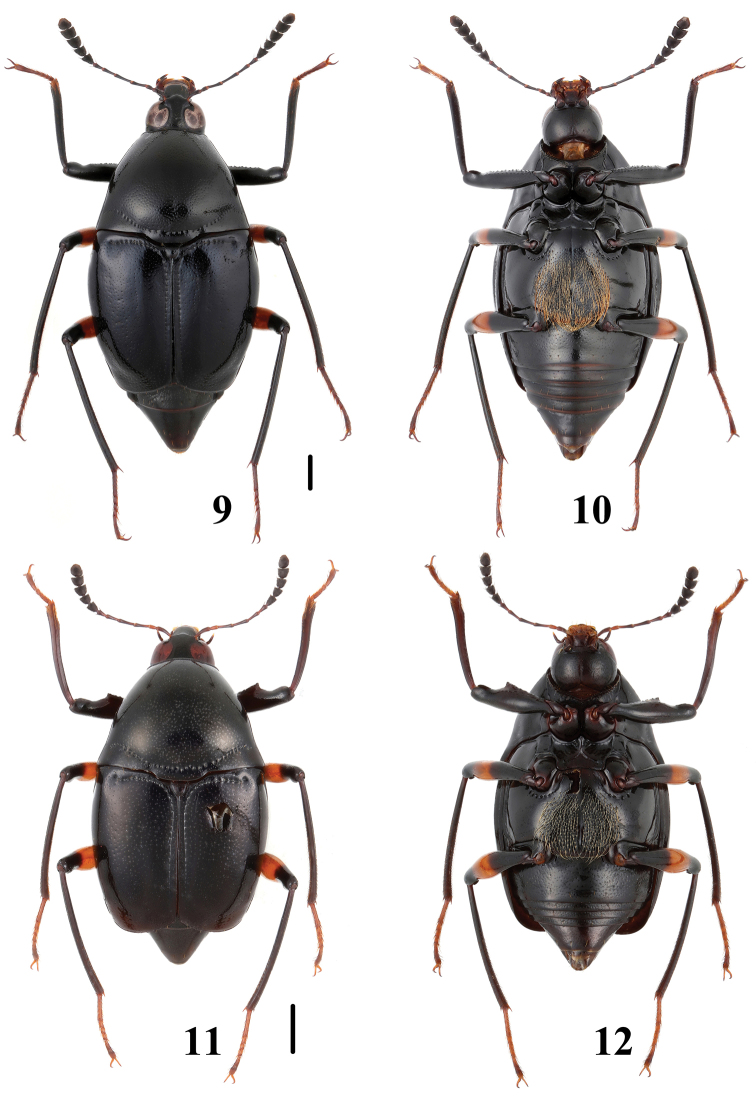
Habitus of *Scaphidium*. **9, 10** *Scaphidium grande* **11, 12** *Scaphidium spinatum*. Scales = 1 mm.

### Scaphidium spinatum

Tang & Li, 2010

http://species-id.net/wiki/Scaphidium_spinatum

Figs 11 , 12 , 74–77 , 163

Scaphidium spinatum Tang & Li, 2010a: 70.

#### Material examined.

**Holotype.** **Anhui:** ♂, Yuexi Couty, Yaoluoping Vil., 17.VII.2007–4.VIII.2007, Ba, Lang & Wang leg. (HBUM). **Paratypes.** 6♂♂12♀♀, same data as for the holotype (1♂1♀ in SHNU, remaining in HBUM).

#### Other material.

**Anhui:** 3♂♂9♀♀, Yuexi County, Yaoluoping, alt. 1050–1650m, 17–21.VI.2013, Dai & Peng leg. (SHNU).

#### Distribution.

China (Anhui).

#### Remarks.

The species can be distinguished from it allies by a spine on the male profemur.

### Scaphidium crypticum sp. n.

http://zoobank.org/DF2BCA13-FB3A-4656-A697-36F1906E4191

http://species-id.net/wiki/Scaphidium_crypticum

Figs 13–16 , 78–81 , 151 , 152

#### Type material.

**Holotype.** **Zhejiang:** ♂, Longquan City, Fengyangshan, alt. 1100 m, 5.VI.2008, W.-X. Bi leg. “Holotype / *Scaphidium crypticum*/ Tang & Li” [red handwritten label] (SHNU). **Paratypes. Zhejiang:** 3♂♂, same data as for the holotype (SHNU); 1♀, Qingyuan County, Baishanzu, 8.VII.2009, Z.-W. Yin leg. (SHNU). **Fujian:** 2♂♂3♀♀, Wuyishan City, Guadun Vil., 27°44'N, 117°38'E, alt 1100–1500m, 25–29.V.2012, Peng & Dai leg. (1 pair in MHNG, remaining in SHNU); 1♂, Wuyishan City, Guadun Vil., 27°44'27"N, 117°37'57"E, alt 1200–1300m, 29.V.2012, X.-B. Song leg. (SHNU). **Jiangxi:** 2♂♂1♀, Yichun City, Fengxin County, Baizhang Vil., 28°41'18"N, 114°46'13"E, alt. 840–860m, 15.VII.2013, Hu & Lv leg. (SHNU). **Guangxi:** 1♂, Shangsi County, Shiwandashan, alt. 300–500m, 4.V.2011, L. Tang leg. (SHNU); 1♀, Lingui County, Huaping N. R., alt. 1200m, 13.VII.2011, Z. Peng leg. (SHNU); 1♀, Jinxiu County, 16km away, alt. 900m, 29.VII.2011, Z. Peng leg. (SHNU).

#### Description.

BL: 4.4–5.1 mm.

Body reddish-yellow. Frons with a triangular blackish median fascia (Fig. 16) on vertex. Antennal club (Fig. 80) blackish with terminal segment slightly lighter in basal 2/3 and distinctly lighter in apical 1/3. Pronotum with two longitudinal black fasciae. Each elytron with one small black humeral spot, one large black median fascia and blackish suture. Prohypomera blackish along the inner side. Prosternum, mesoventrite and metaventrite black. Abdominal sternite III widely black on median portion. Trochanter more or less blackish. Femora blackish except ventral side reddish yellow. A specimen from Guangxi (Fig. 15) with slightly variable coloration: prohypomera and prosternum yellow, mesoventrite and trochanter reddish, femora yellow.

**Figures 13–17.**
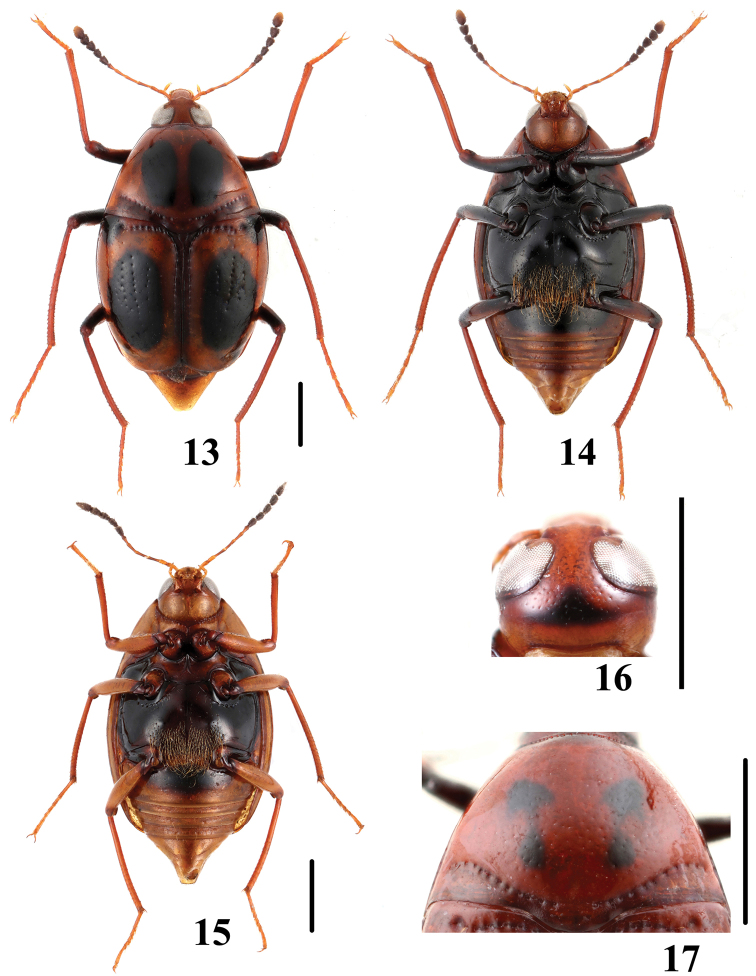
*Scaphidium* spp. **13, 14** habitus of *Scaphidium crypticum* (Zhejiang) **15** habitus of *Scaphidium crypticum* (Guangxi) **16** head of *Scaphidium crypticum* **17** pronotum of *Scaphidium varifasciatum*. Scales = 1 mm.

Frons finely and sparsely punctate, punctures of vertex coarser than those on remaining surface.

Pronotum slightly raised above elytra. Antebasal puncture row impressed, interrupted at middle, with punctures coarse and regular; discal punctation similar to that of vertex, intervals as broad as 1.5 to 2 puncture diameter.

Elytra with disc slightly impressed apically, basal and sutural stria rows impressed; discal punctation similar to that of pronotum except that on apical impression which is very coarse and dense; each elytron with 3 long discal puncture rows consisting of very coarse punctures, 1^st^ row more or less indistinct, 2^nd^ and 3^rd^ rows distinct; basal stria row with punctures slightly coarser than those forming pronotal antebasal row, sutural stria puncture row relatively fine.

Prohypomera slightly uneven, with moderately coarse and very shallow punctures.

Mesepisterna finely, sparsely and very shallowly punctate.

Abdominal tergites with relatively fine and sparse punctures. Sternite III with distinct micropunctures on median portions, remaining sternites on basal half with dense microsculpture consisting of micropunctures.

Legs relatively long, mesotibiae and metatibiae moderately curved.

Male. Metaventrite (Fig. 14) impressed at middle, with long and suberect pubescence. Protibiae (Fig. 81) almost straight with small tubercles along ventral sides. Median lobe of aedeagus (Fig. 78) with sclerotized internal sac (Fig. 79) consisting of two apical sclerotized rods, x-shaped median sclerite and two basal sclerotized rods.

#### Distribution.

China (Zhejiang, Fujian, Jiangxi, Guangxi).

#### Remarks.

The species is similar to *Scaphidium biwenxuani* in its fascia pattern, but can be distinguished from the latter by the elytra lacking the inner basal black dot and the posterior portion of the head in having a black spot.

#### Etymology.

The Latin adjective “crypticum” means mysterious.

### Scaphidium varifasciatum sp. n.

http://zoobank.org/73435331-5B55-4AFC-A858-DAB6CABA2F1A

http://species-id.net/wiki/Scaphidium_varifasciatum

Figs 17 –19 , 82–85

#### Type material.

**Holotype.** **Zhejiang:** ♂, Lin’an City, West Tianmushan, alt. 1000 m, 18.VIII.2011, L. Tang leg. “Holotype / *Scaphidium varifasciatum* / Tang & Li” [red handwritten label] (SHNU). **Paratypes. Zhejiang:** 2♂♂2♀♀, same data as for the holotype (1 pair in MHNG, remaining in SHNU); 1♂, Anji City, Longwangshan, 30°27'N, 119°26'E, alt. 300–500m, 7.VI.2012, Hu & Yin leg. (SHNU); 1♂, Anji City, Tonghanggang, 30°24'N, 119°26'E, alt. 1480m, 10.VI.2012, J.-Q. Zhu leg. (SHNU). **Anhui:** 1♂, Yuexi County, Yaoluoping N. R., Ximianzi Vil., 30°58'55"N, 116°3'49"E, alt. 1050m, 21.VI.2013, Dai & Peng leg. (SHNU).

#### Description.

BL: 3.7–4.6 mm.

**Figures 18–21.**
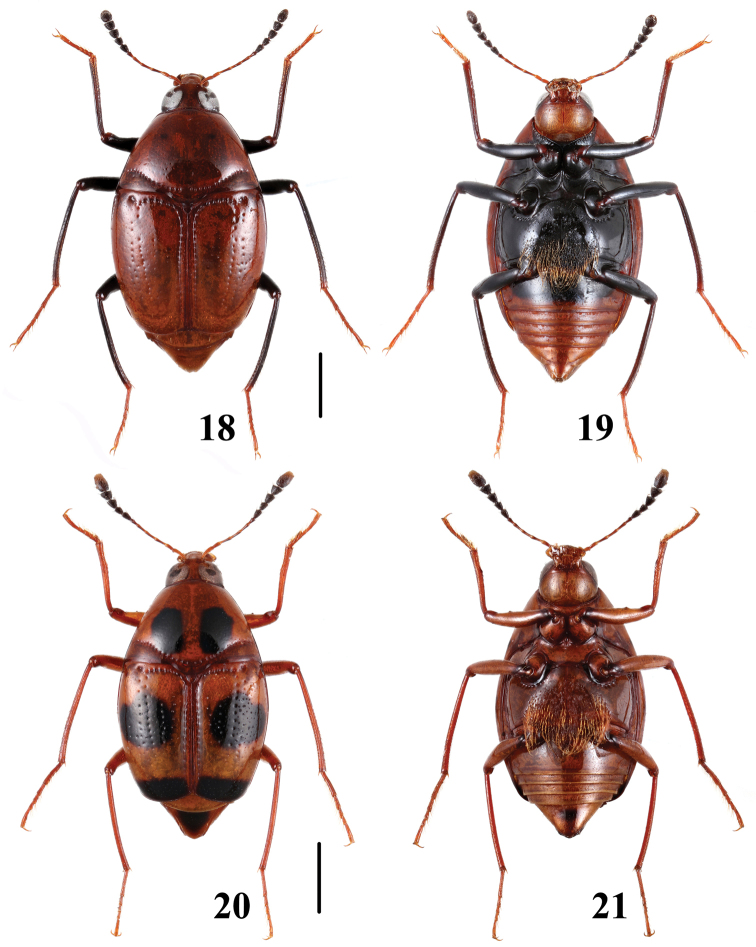
Habitus of *Scaphidium*. **18, 19** *Scaphidium varifasciatum* **20, 21** *Scaphidium sauteri*. Scales = 1 mm.

Body reddish. Antennal club (Fig. 84) blackish with terminal segment slightly lighter in basal half and distinctly lighter in apical half. Pronotal fasciae variable, with pair of faint dots (Fig. 18) to two pairs of black dots, the apical dots sometimes connected to basal dots by black stripe (Fig. 17). Inner halves of prohypomera blackish. Prosternum, mesoventrite and metaventrite black. Abdominal sternite III widely black on median portion. Trochanters more or less blackish. Femora varied from reddish-brown to blackish and tibia varied from reddish to dark brown.

Frons finely and sparsely punctate, punctures on vertex coarser than those on remaining surface.

Pronotum slightly raised above elytra. Antebasal puncture row impressed, interrupted at middle, with punctures coarse and regular; discal punctation similar to that of vertex, puncture intervals as broad as 1.5 to 3 puncture diameter.

Elytra with disc slightly impressed apically, basal and sutural stria rows impressed; discal punctation similar to that of pronotum except that on apical impression which is very coarse and dense; each elytron with 3 long discal puncture rows consisting of very coarse punctures, 1^st^ row short and more or less indistinct, 2^nd^ and 3^rd^ rows long and distinct, rarely the presence of an additional row between 1st row and sutural stria rows may be recognized; basal stria row with punctures slightly coarser than those forming pronotal antebasal row, sutural stria puncture row relatively fine.

Prohypomera slightly uneven, with few indistinct punctures on posterior portion.

Mesepisterna smooth.

Abdominal tergites with relatively fine and sparse punctures. Sternite III with distinct micropunctures on median portions, remaining sternites on basal half with dense microsculpture consisting of micropunctures.

Legs relatively long, mesotibiae and metatibiae moderately curved.

Male. Metaventrite (Fig. 19) impressed at middle, with long and suberect pubescence. Protibiae (Fig. 85) almost straight with small tubercles along ventral sides. Median lobe of aedeagus (Fig. 82) with sclerotized internal sac (Fig. 83) consisting of two apical sclerotized rods, x-shaped median sclerite and two basal sclerotized rods.

#### Distribution.

China (Zhejiang, Anhui).

#### Remarks.

The species is distinctive for its unique coloration.

#### Etymology.

The Latin adjective “varifasciatum” refers to the variable fasciae of the pronotum.

### Scaphidium sauteri

Miwa & Mitono, 1943

http://species-id.net/wiki/Scaphidium_sauteri

Figs 20 , 21 , 86–89

Scaphidium sauteri Miwa & Mitono, 1943: 529.

#### Material examined.

**Fujian:** 1♂1♀, Wuyishan City, Guadun Vil., 27°43'59"N, 117°39'3"E, alt. 1000–1100m, 27.V.2012, X.-B. Song leg. (SHNU); 1♂: ibidem, 27°44'25"N, 117°38'9"E, alt. 1200–1300m, 26.V.2012, X.-B. Song leg. (SHNU). **Anhui:** 1♀, Yuexi County, Yaoluoping N. R., Ximianzi Vil., 30°58'55"N, 116°3'49"E, alt. 1050m, 21.VI.2013, Dai & Peng leg. (SHNU). **Zhejiang:** 1♂, Kaihua County, Gutianshan, 29°14'N, 118°6'E, alt. 320m, 20.V.2013, J.-Y. Hu leg. (SHNU); ibidem, 29°14'N, 118°8'E, alt. 400–500m, 19.VI.2013, Lv & Xie leg. (SHNU). **Jiangxi:** 1♂3♀♀, Jinggangshan City, Ciping, alt. 850m, 18.X.2010, Peng, Zhai & Zhu leg. (SHNU). **Guangdong:** 1♀, Shaoguan City, Ruokeng, 30.VIII.2008, B.-P. Huang leg. (SHNU). **Guangxi:** 1♀, Lingui County, Huaping N. R., 28.X.2009, Y. Liu leg. (SHNU); 1♀, Lingui County, Huaping N. R., Anjiangping, alt. 1200m, 13.VII.2011, Z. Peng leg. (SHNU)

#### Distribution.

China (Zhejiang, Anhui, Fujian, Jiangxi, Guangdong, Guangxi, Taiwan).

#### Remarks.

The type depository of the species is TARI and the photo of the type specimen was sent to us by Dr. Chi-Feng Lee. Each pronotal fascia of the type extends to lateral side basally along the antebasal puncture row, which is absent in specimens from mainland China; this is temporarily considered as intra-specific variability. In the habitus photo, there is a black spot on the vertex similar to that in Fig. 16, which is covered by the pronotum.

### Scaphidium formosanum

Pic, 1915

http://species-id.net/wiki/Scaphidium_formosanum

Figs 22–25 , 90–93

Scaphidium formosanum Pic, 1915a: 36; Löbl 1999: 708.

#### Material examined.

**Jiangxi:** 1♀, Kiangsi, 1929, J Sedlacek Collection (MHNG). **Fujian:** 1♀, Wuyishan City, Guadun Vil., 27°43'1"N, 117°39'26"E, alt. 1000–1100m, 31.V.2012, X.-B. Song leg. (SHNU). **Guangdong:** 1♀, Ruyuan County, Nanling N. R., alt. 1050m, 15.VII.2012, L. Ning leg. (SHNU). **Yunnan:** 1♀, Baoshan City, Baihualing, 25°16'46"N, 98°47'20"E, alt. 1350–1450m, 22.IV.2013, Peng & Dai leg. (SHNU). **Guangxi:** 1♂, Shangsi County, Shiwandashan N. R., alt. 300–700m, 24.IV.2011, Zhai, Peng & Zhu leg. (SHNU); 3♂♂2♀♀, Jinxiu County, Shengtangshan, alt. 700m, 28.VII.2011, Z. Peng leg. (SHNU); 1♂, Damingshan, 23°23'N, 108°29'E, alt. 1200–1300m, 30.VII.2012, Hu & Song leg. (SHNU). **Taiwan:** 1♂, Fuliosha, 09.VIII. (NMPC). **Hainan:** 1♂1♀, Changjiang County, Bawangling, alt. 1000m, 10.IV.2010, B.-P. Huang leg. (SHNU); 6♂♂1♀, Ledong County, Jianfengling N. R., alt. 1000m, 18.V.2011, W.-X. Bi leg. (SHNU); 1♂1♀, Wuzhishan City, Shuiman, Wuzhishan, alt. 700m, 18.IV.2012, Peng & Dai leg. (SHNU); 9♂♂1♀, Ledong County, Jianfengling N. R., Mingfenggou, 18°44'N, 108°50'E, alt. 950m, 29.IV.2012, Peng & Dai leg. (SHNU)

#### Distribution.

China (Jiangxi, Fujian, Guangdong, Yunnan, Guangxi, Hainan, Taiwan).

#### Remarks.

These are new province records to Fujian, Guangdong, Yunnan, Guangxi, Hainan. Specimens from Hainan (Figs 24, 25) have smaller black marks, especially on the ventral side and legs, the second inner black spot is absent in approximately half of the specimens. However, no differences are found in sexual characters, suggesting that the Hainan population may represent a new subspecies. The species is very similar to *Scaphidium baconi* Pic, 1915 (see Pic 1915b) distributed in Nepal, India, Thailand, and Vietnam. Reliable distinguishing characters between them are unknown, and the validity of *Scaphidium baconi* is suspicious. The species is also similar to *Scaphidium carinense* Achard; for differences see remarks below.

**Figures 22–25.**
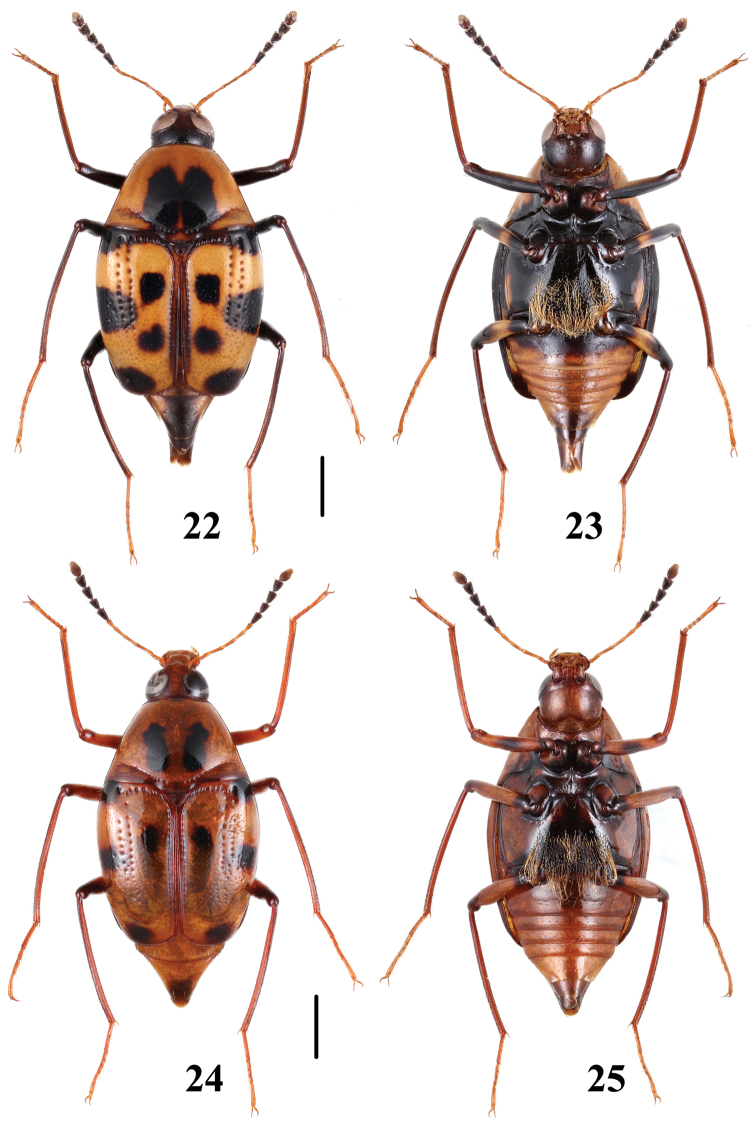
Habitus of *Scaphidium formosanum*. **22, 23** (Guagnxi) **24, 25** (Hainan). Scales = 1 mm.

### Scaphidium carinense

Achard, 1920: 239

http://species-id.net/wiki/Scaphidium_carinense

**Figures 26–29.**
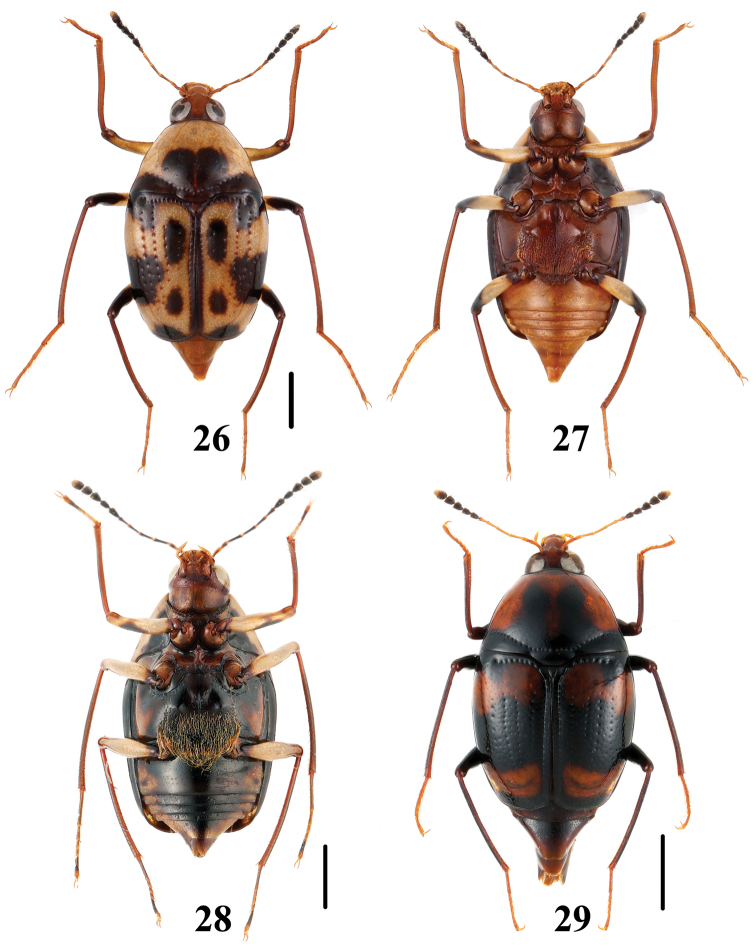
Habitus of *Scaphidium*. **26–28** *Scaphidium carinense* **29** *Scaphidium sinense* (Zhejiang). Scales = 1 mm.

Figs 26 , 27 , 94–97 , 153

Scaphidium carinense Achard, 1920a: 239; Tang and Li 2013: 174.

#### Material examined.

**Lectotype.** **Myanmar:** ♀, Carin Cheba. (NMPC).

#### Other material.

**Hubei:** 2♂♂5♀♀, Wufeng County, Houhe N. R., 30°5'7"N, 110°33'11"E, alt. 1200m, 9.VII.2013, Dai, Peng & Xie leg. (SHNU). **Fujian:** 12♂♂8♀♀, Guadun, Wuyishan, 27°44'N, 117°38'E, alt. 1000–1400m, 26–29.V.2012, Peng, Dai & Song leg. (SHNU). **Sichuan:** Shimian County, Liziping, Zima Vil., 28°59'N, 102°16'E, alt. 1800m, 16.VII.2012, Peng, Dai & Yin leg. (SHNU). **Guangxi:** 56♂♂17♀♀, Lingui County, Huaping N. R., Anjiangping, alt. 1200–1300m, 13–18.VII.2011, Z. Peng leg. (SHNU); 16♂♂5♀♀, Jinxiu County, l6km away, alt. 900m, 29–31.VII.2011, Z. Peng leg. (SHNU); 10♂♂6♀♀, Shangsi County, Shiwandashan, alt. 300–700m, 24.25.IV.2011, Zhai, Peng & Zhu leg. (SHNU). **Yunnan:** 10♂♂3♀♀, Menla, Wangtianshu, alt. 600m, 6.VI.2009, Wen-Xuan Bi leg. (SHNU). **Hainan:** 2♂♂5♀♀, Lingshui County, Diaoluoshan, 18°43'N, 109°51'E, alt. 1000m, 24.IV.2012, Peng & Dai leg. (SHNU); 13♂♂12♀♀, Wuzhishan City, Wuzhishan, 18°44'N, 108°50'E, alt. 950m, 29.IV.2012, Peng & Dai leg. (SHNU).

#### Distribution.

China (Hubei, Fujian, Sichuan, Guangxi, Yunnan, Hainan), Myanmar.

#### Remarks.

This is a new province record to Hubei. The species is similar to *Scaphidium formosanum* Pic and can be distinguished from the latter by its elytra bearing two additional inner puncture rows between the long outer puncture rows and the sutural puncture row.

### Scaphidium sinense

Pic, 1954

http://species-id.net/wiki/Scaphidium_sinense

Figs 28 –33 , 98–101 , 154

Scaphidium sinense Pic, 1954: 57; Löbl 1992: 583; Löbl 1999: 711; He et al. 2008b: 59.

#### Material examined.

**Zhejiang:** 1♂, Lin’an City, Tianmushan, alt. 1100m, 2.V.2005, W.-X. Bi leg. (SHNU); 1♂, ibidem, alt. 1200m, 6.V.2007, W.-X. Bi leg. (SHNU); 1♂, ibidem, 15.VIII.2007, Y.-X. Wu leg. (SHNU); 4♂♂4♀♀, ibidem, alt. 1000m, 7–15.VIII.2010, L. Tang leg. (SHNU); 1♂, ibidem, alt. 250m, 15.VIII.2010, L. Tang leg. (SHNU); 1♂7♀♀, ibidem, alt. 1000–1200m, 18–21.VIII.2011, L. Tang leg. (SHNU); 1♂, ibidem, 30°19'10"N, 119°26'51"E, alt. 410m, 21.X.2013, Tang leg. (SHNU); 1♂, Lin’an City, Pingxi, 30°23'N, 119°28'E, alt. 1000–1100m, 9.VI.2012, J.-Q. Zhu leg. (SHNU); 1♂, Qingyuan County, Baishanzu, alt. 900–1400m, Hu, Tang & Zhu leg. (SHNU); 1♂, Kaihua County, Gutianshan, 29°15'N, 118°8'E, 21.VI.2013, X.-B. Song leg. (SHNU). **Jiangxi:** 1♂, Yushan County, Sanqingshan, Fenshui, alt. 400m, 12.X.2010, Peng, Zhai & Zhu leg. (SHNU); 1♀, Jiulianshan N. R., 27–28.VI.2012, Li leg. (SHNU); 1♀, Yichun City, Mingyueshan Park, 27°35'32"N, 114°17'13"E, alt. 1200–1600m, 12.VII.2013, Song, Yin & Yu leg. (SHNU); 3♂♂5♀♀, Luxi County, Yangjialing, 27°35'3"N, 114°15'2"E, alt. 820m, 15.VII.2013, Song, Yin & Yu leg. (SHNU); 1♀, Pingxiang City, Gaozhou County, Gaotianyan, 27°23'51"N, 114°0'54"E, alt. 1025m, 23.VII.2013, Song, Yin & Yu leg. (SHNU). **Hunan:** 3♀♀, Yanling County, Taoyuandong Park, 26°29'14"N, 114°0'42"E, alt. 770m, 16.VII.2013, Dai, Peng & Xie leg. (SHNU); 3♂♂3♀♀, Liuyang City, Daweishan, 28°25'28"N, 114°4'52"E, alt. 830m, 22.VII.2013, Dai, Peng & Xie leg. (SHNU); 2♂♂7♀♀, ibidem, 28°25'37"N, 114°7'43"E, alt. 1430m, 21.VII.2013, Dai, Peng & Xie leg. (SHNU). **Guangxi:** 20♂♂20♀♀, Shangsi County, Shiwandashan, alt 300–500m, Zhai, Peng, Zhu & Tang leg. (SHNU); 5♂♂17♀♀, Lingui County, Huaping N. R., Anjiangping, alt. 1200–1300m, 13–16.VII.2011, Ma, Chen, Zhu, Peng, Tang & He leg. (SHNU); 1♀, Jinxiu County, 16km away, alt. 900m, 31.VII.2011, Z. Peng leg. (SHNU).

#### Distribution.

China (Zhejiang, Fujian, Jiangxi, Hunan, Guangxi).

#### Remarks.

**Figures 30–33.**
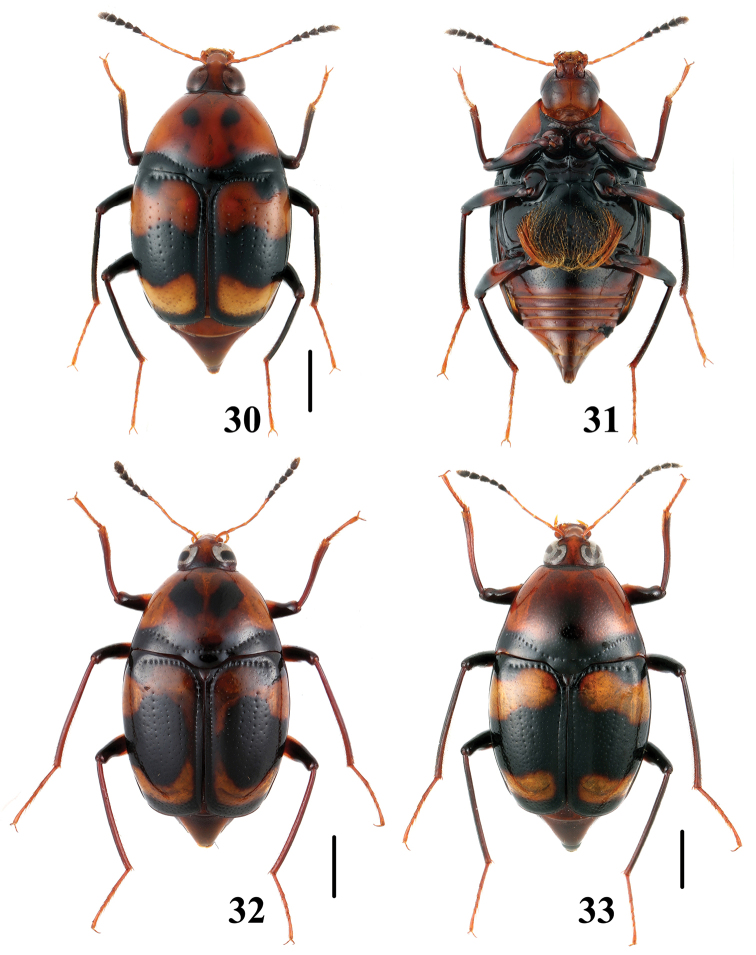
Habitus of *Scaphidium sinense*. **30, 31** (Zhejiang) **32** (Guangxi, Huaping) **33** (Guangxi, Shiwandashan). Scales = 1 mm.

These are new records to Jiangxi, Hunan, Guangxi. The fascia pattern of pronotum is variable (Figs 29, 30, 32, 33) and the usually bicolored legs are entirely darkened in some individuals. The color pattern is related to geographical populations as suggested by following: approximately half of the specimens from Hunnan, Jiangxi and North Guangxi have the sub-basal and subapical fasciae of the elytra joined along the suture (Fig. 32); in specimens from Shiwandashan in South Guangxi (Fig. 33), the pronotal fasciae tends to indistinct, the sub-basal and subapical fasciae of elytra are round on their inner corners; the body size of the Guangxi population is larger on average (4.4–5.9 mm) than that of other populations (4.1–5.3 mm). The species is similar to *Scaphidium harmandi* Achard, 1920 (see Achard 1920b) and differs in the bicolored legs which are entirely reddish in *Scaphidium harmandi*, and darkened terminal antennal segment which is entirely yellowish in *Scaphidium harmandi*.

### Scaphidium delatouchei

Achard, 1920

http://species-id.net/wiki/Scaphidium_delatouchei

Figs 34–36 , 102–105 , 155

Scaphidium delatouchei Achard, 1920c: 210; Löbl 1999: 708.

#### Material examined.

**Zhejiang:** 2♂♂, Lin’an City, West Tianmushan, alt. 250m, 15.VIII.2010, L. Tang leg. (SHNU); 1♂, ibidem, alt. 1000m, 7.VIII.2010, L. Tang leg. (SHNU); 1♀, ibidem, alt. 1000–1200m, 18.VIII.2011, L. Tang leg. (SHNU); 1♂, Anji County, Tonghanggang, 30°24'N, 119°26'E, alt. 1480m, 10.VI.2012, J.-Q. Zhu leg. (SHNU); 1♀, ibidem, 30°25'N, 119°26'E, alt. 1100m, 11.VI.2012, Hu & Yin leg. (SHNU); 1♂, Anji County, Longwangshan, Qianmutian, 30°24'N, 119°21'E, alt. 1050–1250m, 8.VI.2012, Hu & Yin leg. (SHNU); 1♂1♀, ibidem, 30°23'59"N, 119°26'26"E, alt. 1350m, 14.V.2013, X.-B. Song leg. (SHNU). **Hunan:** 1♂, Pingliang County, Mufushan, 28°58'18"N, 113°49'55"E, alt. 850m, 25.VII.2013, Dai, Peng & Xie leg. (SHNU); 1♂1♀, Taoyuandong Park, 26°29'14"N, 114°0'42"E, alt. 770m, 16.VII.2013, Dai, Peng & Xie leg. (SHNU); 2♀♀, Liuyang City, Daweishan, 28°25'37"N, 114°7'43"E, alt. 1430m, 21.VII.2013, Dai, Peng & Xie leg. (SHNU). **Hubei:** 1♀, Wufeng County, Houhe N. R., 37°5'9"N, 110°33'5"E, alt. 1160m, 8.VII.2013, Dai, Peng & Xie leg. (SHNU). **Anhui:** 1♂2♀♀, Yuexi County, Yaoluoping, alt. 1050–1430m, 17–21.VI.2013, Dai & Peng leg. (SHNU). **Sichuan:** 1♂,Qingchengshan, alt. 1100m, 8.VIII.2009, Tang & He leg. (SHNU); 1♀, Tianquan County, Lianglu, Shaochaigou, alt. 1530m, 11.IX.2011, W.-X. Bi leg. (SHNU); 1♂, Tianquan County, Laba River, 30°4'N, 102°25'E, alt. 1400m, 12.VII.2012, Peng, Dai & Yin leg. (SHNU). **Yunnan:** 2♀♀, Gongshan County, Bingzhongluo, Niwaluo, alt. 1862m, 28°3'287"N, 98°56'995"E, 15.VIII.2006, Y. Liu leg. (SHNU); 7♂♂6♀♀, Binchuan County, Jizushan, alt. 2400m, 18.VII.2010, L. Tang leg. (SHNU); 2♂♂2♀♀, Lijiang City, Hutiaoxia, alt. 1700m, 1.VIII.2010, X.-B. Song leg. (SHNU); 1♂, Baoshan City, Baihuailing, 25°16'46"N, 98°47'20"E, alt. 1350–1450m, 22.IV.2013, Peng & Dai leg. (SHNU). **Guangxi:** 1♀, Damingshan, 23°23'N, 108°29'E, alt. 1200–1300m, 30.VII.2012, Hu & Song leg. (SHNU).

#### Distribution.

China (Zhejiang, Hunan, Hubei, Anhui, Sichuan, Yunnan, Guangxi, Guangdong).

#### Remarks.

These are new records to Zhejiang, Hunan, Hubei, Anhui, Sichuan and Guangxi. Most specimens from Jizushan and Hutiaoxia of Central Yunnan have the subapical fascia of elytra reduced to a spot (Fig. 36). The species is distinctive by its coloration and body size.

**Figures 34–37.**
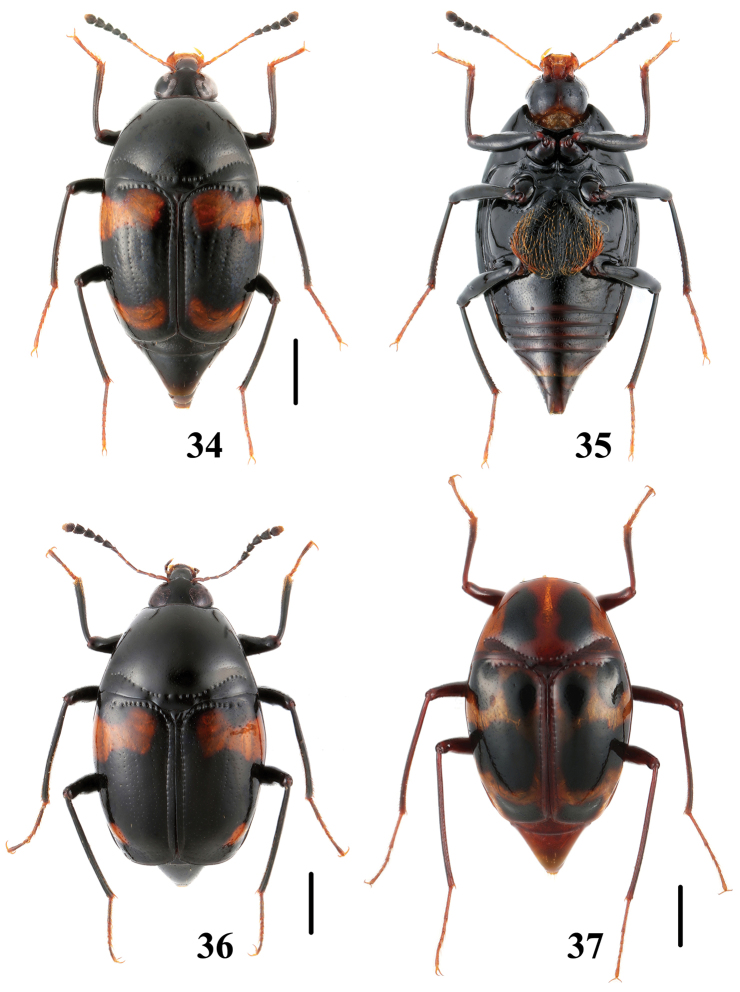
Habitus of *Scaphidium*. **34–36** *Scaphidium delatouchei* **37** *Scaphidium biwenxuani* (Yunnan). Scales = 1 mm.

### Scaphidium biwenxuani

He, Tang & Li, 2008

http://species-id.net/wiki/Scaphidium_biwenxuani

Figs 37 –39 , 106–109 , 156–162

Scaphidium biwenxuani He, Tang & Li, 2008a: 178; Tang and Li 2013: 180.

#### Material examined.

**Holotype.** **Zhejiang:** ♂, Anji County, Longwangshan, alt. 950–1200m, 25.IV.2006, Bi & Tang leg. (SHNU).

#### Paratypes.

**Zhejiang:** 5♂♂2♀♀, same data as for the holotype (SHNU); 1♂1♀, Lin’an City, Tianmushan, alt. 1100m, 24.VIII.2006, Bi & Tang leg. (SHNU); 1♂1♀, ibidem, 18.IV.2007, Y.-X. Wu leg. (SHNU); 1♂, ibidem, 15.VIII.2007, H. Huang leg. (SHNU); 1♀, ibidem, 2-VIII-2006, X.-B. Song leg. (SHNU). **Anhui:** 4♀♀, Guniujiang, alt. 950–1050m, 28.IV.2005, Hu & Tang leg. (SHNU).

#### Other material.

**Zhejiang:** 10♂♂13♀♀, Lin’an City, Tianmushan, alt. 1000m, 7.VIII.2010, L. Tang leg. (SHNU); 2♂♂, ibidem, alt. 1000m, 19. III.2009, M. Jin leg. (SHNU); 9♂♂8♀♀, ibidem, alt. 1000–1200m, 18–21.VIII.2011, L. Tang leg. (SHNU); 1♂5♀♀, ibidem, 12.V.2012, Li leg. (SHNU); 5♂♂8♀♀, Anji County, Longwangshan, alt. 1000–1500m, 8–10.VI.2012, Zhu, Hu & Yin leg. (SHNU); 26♂♂18♀♀, ibidem, alt. 1300–1500m, 14–19.V.2013; Tang, Dai & Peng leg. (SHNU); 1♂1♀, Qingyuan County, Baishanzu, alt. 1500m, 23.IX.2008, L. Tang leg. (SHNU). **Sichuan:** 1♂2♀♀, Tianquan County, Laba River, 30°4'N, 102°25'E, alt. 1400m, 12.VII.2012, Peng, Dai & Yin leg. (SHNU). **Hunan:** 1♂, Yanling County, Taoyuandong Park, 26°29'14"N, 114°0'42"E, alt. 770m, Dai, Peng & Xie leg. (SHNU). **Hubei:** 1♂4♀♀, Wufeng County, Houhe N. R., 30°5'7"N, 110°33'11"E, alt. 1200m, 9.VII.2013, Dai, Peng & Xie leg. (SHNU); 1♀, ibidem, 3.VIII.2013, H. Huang leg. (SHNU). **Jiangxi:** 1♂4♀♀, Yichun City, Mingyueshan Park, 27°35'32"N, 114°17'13"E, alt. 1200–1600m, 12.VII.2013, Song, Yin & Yu leg. (SHNU); 1♀, ibidem, 27°35'44"N, 114°16'26"E, alt. 1140m, 23.X.2013, Song, Yin & Yan leg. (SHNU). **Guizhou:** 1♀, Leishan County, Leigongshan, Lianhuaping, 13.VIII.2011, H. Xu leg. (SHNU); 1♀, Suiyang County, Kuankuoshui N. R., Gongtonggou, alt. 1550m, 9.VI.2010, Yin, Zhai & Liu leg. (SHNU). **Guangxi:** 3♂♂, Jinxiu County, Shengtangshan, alt. 1900m, 25.VII.2011, Z. Peng leg. (SHNU); 2♀♀, Jinxiu County, Yinshan Baohuzhan, alt. 1200m, 23.VII.2011, Zhu, Hu & Song leg. (SHNU); 1♀, Shangsi County, Shiwandashan, alt. 300–700m, 23.IV.2011, Zhai, Peng & Zhu leg. (SHNU); 1♂, Lingui County, Huaping N. R., Anjiangping, alt. 1200m, 13.VII.2011, Tang & He leg. (SHNU); 1♀, Damingshan, 23°23'N, 108°29'E, alt. 1200–1300m, 30.VII.2012, Hu & Song leg. (SHNU). **Yunnan:** Daweishan N. R., Yuping, alt. 2000m, 20.V.2009, W.-X. Bi leg. (SHNU).

#### Distribution.

China (Zhejiang, Anhui, Hunan, Hubei, Jiangxi, Guizhou, Sichuan, Yunnan, Guangxi).

#### Remarks.

These are new records to Hunan, Hubei, Jiangxi, Sichuan and Yunnan. The pattern of the fasciae in this species is rather invariable though it is distinctly bold in the specimen from Yunnan (Fig. 37). The species is similar to *Scaphidium robustum* sp. n., for differences see remarks below.

**Figures 38–41.**
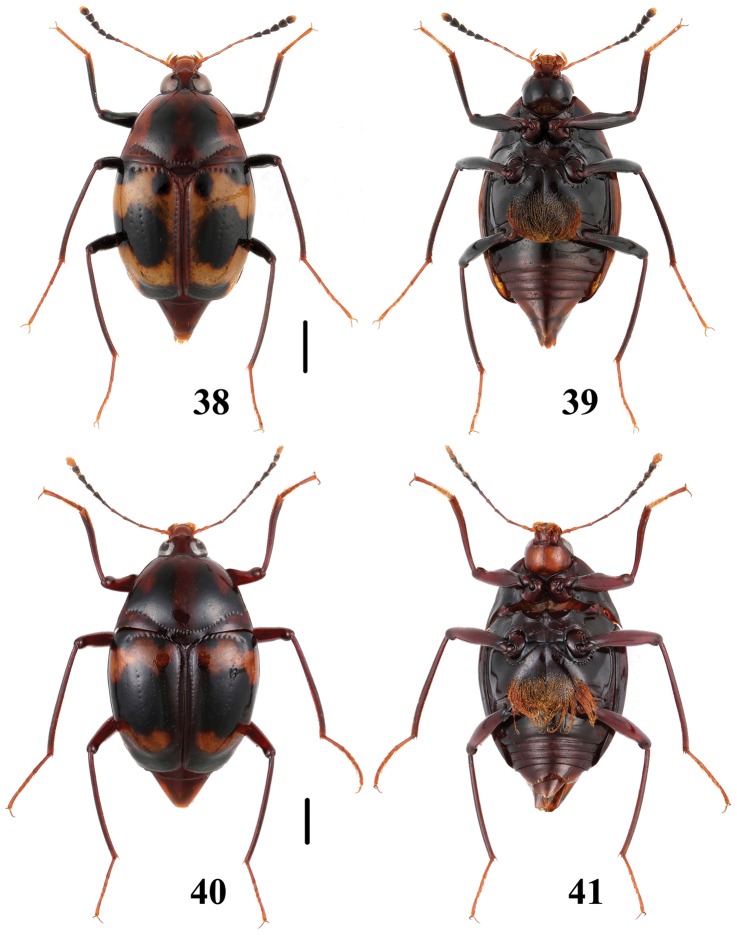
Habitus of *Scaphidium*. **38, 39** *Scaphidium biwenxuani* (Zhejiang) **40, 41** *Scaphidium robustum*. Scales = 1 mm.

### Scaphidium robustum sp. n.

http://zoobank.org/D8B83013-0ED1-47A6-8B66-FBD104B785D7

http://species-id.net/wiki/Scaphidium_robustum

Figs 40 , 41 , 110–113

#### Type material.

**Holotype.** **Fujian:** ♂, Wuyishan City, Guadun Vil., 27°44'N, 117°37'E, alt. 1200–1500m, 28.V.2012, Peng & Dai leg. “Holotype / *Scaphidium robustum* / Tang & Li” [red handwritten label] (SHNU).

#### Paratypes.

**Fujian:** 1♀, same data as for the holotype (SHNU); 1♂3♀♀, ibidem, 27°44'N, 117°38'E, alt. 1100–1500m, 27.V.2012, Dai, Peng & Song leg. (1 pair in MHNG, remaining in SHNU). **Chongqing:** 2♂♂, Nanchuan, Jinfoshan, 29°2'N, 107°11'E, alt. 1000–1300m, 8.IV.2012, H. Huang leg. (SHNU). **Guizhou:** 1♂, Leishan County, Leigongshan, Lianhuaping, 15.IX.2005, Y. Liu leg. (SHNU). **Guangxi:** 1♂, Lingui County, Huaping N. R., Anjiangping, alt. 1400–1700m, 14.VII.2011, Z. Peng leg. (SHNU); 1♀, Jinxiu County, l6km away, alt. 900m, 31.VII.2011, Z. Peng leg. (SHNU); 1♀, Jinxiu County, Shengtangshan, alt. 1200–1400m, 25.VII.2011, Hu & Yin leg. (SHNU); 1♀, Nanning City, Damingshan, alt. 1200m, 21–23.IV.2012, Li leg. (SHNU). **Yunnan:** 1♂, Lushui County, Pianma, 9–11.V.2004, Yang & Liu leg. (HBUM).

#### Description.

BL: 5.7–6.4 mm.

Body mostly reddish-brown with ventral side, except head and sternites, slightly darker. Antennal club (Fig. 112) blackish with terminal segment entirely yellowish. Pronotum with two longitudinal black fasciae, each fascia usually extends laterally basally along the antebasal puncture row, sometimes reduced to longitudinal fascia at middle and separated small lateral spot. Elytra blackish, each with two orange fasciae. Subhumeral fascia tridentate anteriorly and bidentate posteriorly. Subapical fascia curved towards base with apicolateral part extending to apicolateral corner of elytron.

Frons finely, sparsely and very shallowly punctate, punctures of vertex coarser than those of remaining portion.

Pronotum slightly raised above elytra. Antebasal puncture row impressed, continuous in middle, with punctures coarse and regular; discal punctation similar to that of vertex, puncture intervals as broad as 1.5 to 4 puncture diameter.

Elytra with disc almost even apically, basal and sutural stria rows impressed; discal punctation similar to that of pronotum except on coarser and more densely punctuated apical portion; each elytron with one indistinct discal puncture rows consisting of slightly coarser punctures; basal stria row with punctures similar to those forming pronotal antebasal row, sutural stria puncture row relatively fine.

Prohypomera slightly uneven and smooth.

Mesepisterna finely, sparsely and very shallowly punctate.

Abdominal tergites with relatively fine and sparse punctures and very dense micropunctures. Sternite III with distinct micropunctures especially on median portions, remaining sternites with dense microsculpture consisting of micropunctures.

Legs relatively long, mesotibiae and metatibiae moderately curved.

Male. Metaventrite (Fig. 41) impressed at middle, with long and suberect pubescence. Protibiae (Fig. 113) slightly sinuate. Median lobe of aedeagus (Fig. 110) with longitudinal bands well developed, sclerotized internal sac (Fig. 111) consisting of one x-shaped apical sclerite and a complex of basal sclerites.

#### Distribution.

China (Fujian, Chongqing, Guizhou, Guangxi, Yunnan).

#### Remarks.

The new species is similar to *Scaphidium biwenxuani*, but can be distinguished from the latter by its larger and broader body, the smaller inner black dot near scutellum, the slender antennal club and the terminal antennal segment entirely yellowish, while in *Scaphidium biwenxuani* it is yellowish in apical third.

#### Etymology.

The Latin adjective “robustum” refers to the robust body form.

### Scaphidium bayibini sp. n.

http://zoobank.org/5C421CE6-1019-4E21-9BF1-1681FCE7FA5C

http://species-id.net/wiki/Scaphidium_bayibini

**Figures 42–45.**
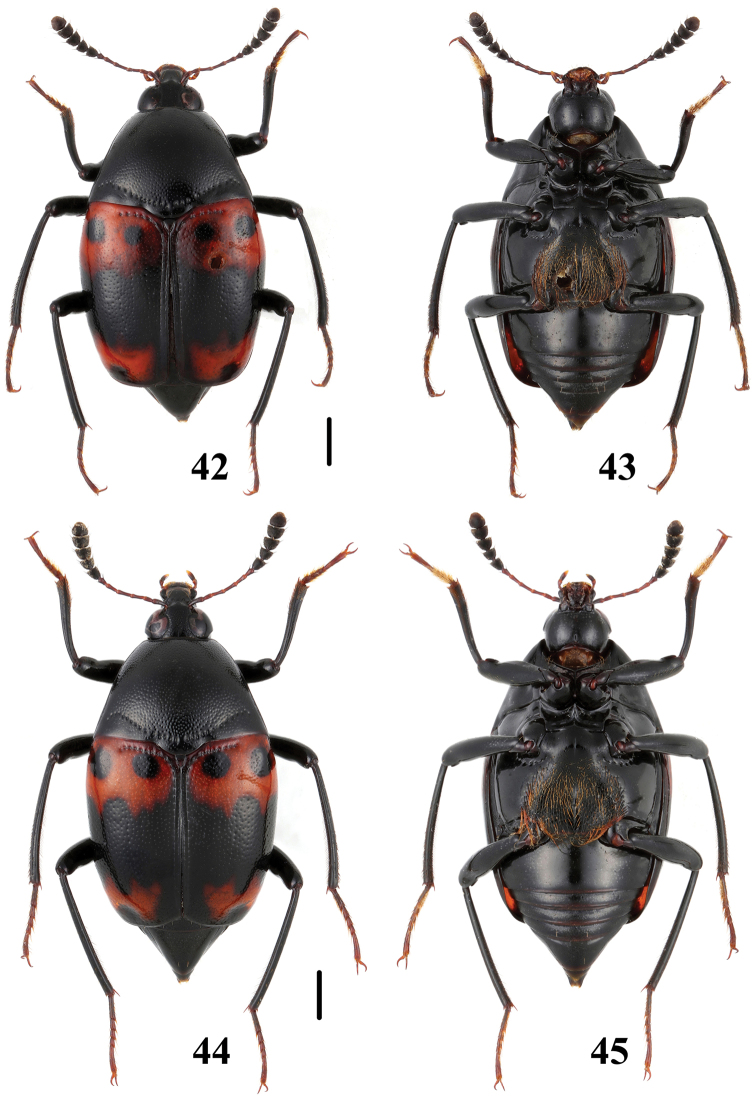
Habitus of *Scaphidium*. **42, 43** *Scaphidium bayibini* **44, 45** *Scaphidium klapperichi*. Scales = 1 mm.

Figs 42 , 43 , 114–117

#### Type material.

**Holotype.** **Anhui:** ♂, Yuexi County, Yaoluoping N. R., Ximianzi Vil., 30°58'55"N, 116°3'49"E, alt. 1050m, 21.VI.2013, Dai & Peng leg. “Holotype / *Scaphidum bayibini* / Tang & Li” [red handwritten label] (SHNU).

#### Paratypes.

**Anhui:** 1♀, same data as for the holotype (SHNU); 1♂, Yuexi County, Yaoluoping, Xiaoqiling, 18–19.VII.2007, Ba, Lang & Wang leg. (HBUM); 1♀, Yuexi County, Yaoluoping Vil., 30.VII–4.VIII.2007, Ba, Lang & Wang leg. (HBUM).

#### Description.

BL: 6.8–7.5 mm.

Body black with antennal segments I–VI and tarsi brownish. Antennal club (Fig. 116) blackish with terminal segment slightly lighter in apical 1/3. Elytra each with two reddish fasciae. Basal fascia large, touching basal and lateral margins and suture of elytron, bidentate posteriorly. Two black dots entirely sealed in basal fascia with inner black dot smaller and separated from basal stria. Subapical fascia tridentate anteriorly and bidentate posteriorly.

Frons coarsely and densely punctate, punctures on vertex denser than those on remaining surface.

Pronotum slightly raised above elytra. Antebasal puncture row impressed, more or less interrupted at middle, with punctures coarse and somewhat elongate; discal punctation coarser than that on vertex, puncture intervals mostly as broad as half puncture diameters.

Elytra with disc slightly impressed apically, basal and sutural stria rows impressed; discal punctation slightly sparser than that of pronotum except that on basal fascia which is distinctly finer and sparser; basal stria row with punctures similar to those forming pronotal antebasal row, sutural stria puncture row relatively fine.

Prohypomera slightly uneven, with relatively fine and very shallow punctures, especially on outer half.

Mesepisterna finely, sparsely and shallowly punctate.

Abdominal tergites with relatively coarse and dense punctures and distinct microsculpture consisting of micropunctures. Sternites with fine and shallow punctures and relatively faint microsculpture consisting of micropunctures.

Legs moderately long, mesotibiae and metatibiae moderately curved.

Male. Metaventrite (Fig. 43) impressed at middle, with long and suberect pubescence. Profemur (Fig. 117) with ventral side moderately expanded, forming two ridges. Protibia (Fig. 117) gradually widened starting from basal 1/3, forming blunt protuberance at widest point, narrowed toward apex. Median lobe of aedeagus (Fig. 114) with distinct longitudinal bands, sclerotized internal sac (Fig. 115) consisting of two apical longitudinal sclerotized rods and two basal transverse sclerotized rods.

#### Distribution.

China (Anhui).

#### Remarks.

The new species is extremely similar to *Scaphidium klapperichi* and can be distinguished by the broader antennal club, the elytra with the inner black dot of basal fascia separated from basal stria, and the distinctive shape of the aedeagal sclerites.

#### Etymology.

This species is named in honor of Mr. Yi-Bin Ba, who firstly collected specimens of the new species.

### Scaphidium klapperichi

Pic, 1954

http://species-id.net/wiki/Scaphidium_klapperichi

Figs 44 , 45 , 118–121 , 164 , 165

Scaphidium klapperichi Pic, 1954: 57; Löbl 1999: 710.

#### Material examined.

**Zhejiang:** 3♂♂, Qingyuan County, Baishanzu, alt. 1500m, 23.IX.2008, L. Tang leg. (SHNU); 1♀, Lin’an City, West Tianmushan, alt. 300m, 26.IV.2008, Z.-W. Yin leg. (SHNU); 1♀, Anji County, Longwangshan, alt. 1000m, 25.V.2012, J.-Q. Zhu leg. (SHNU). **Fujian:** 6♂♂9♀♀, Wuyishan City, Guadun, alt. 1100–1500m, 25–29.V.2012, Peng & Dai leg. (SHNU).

#### Distribution.

China (Zhejiang, Fujian).

#### Remarks.

This is new record to Zhejiang. The species is extremely similar to *Scaphidium bayibini* and can be distinguished by the slender antennal club, the elytra each with the inner black dot of basal fascia touching basal stria and the different shape of the aedeagal sclerites.

### Scaphidium stigmatinotum

Löbl, 1999

http://species-id.net/wiki/Scaphidium_stigmatinotum

**Figures 46–49.**
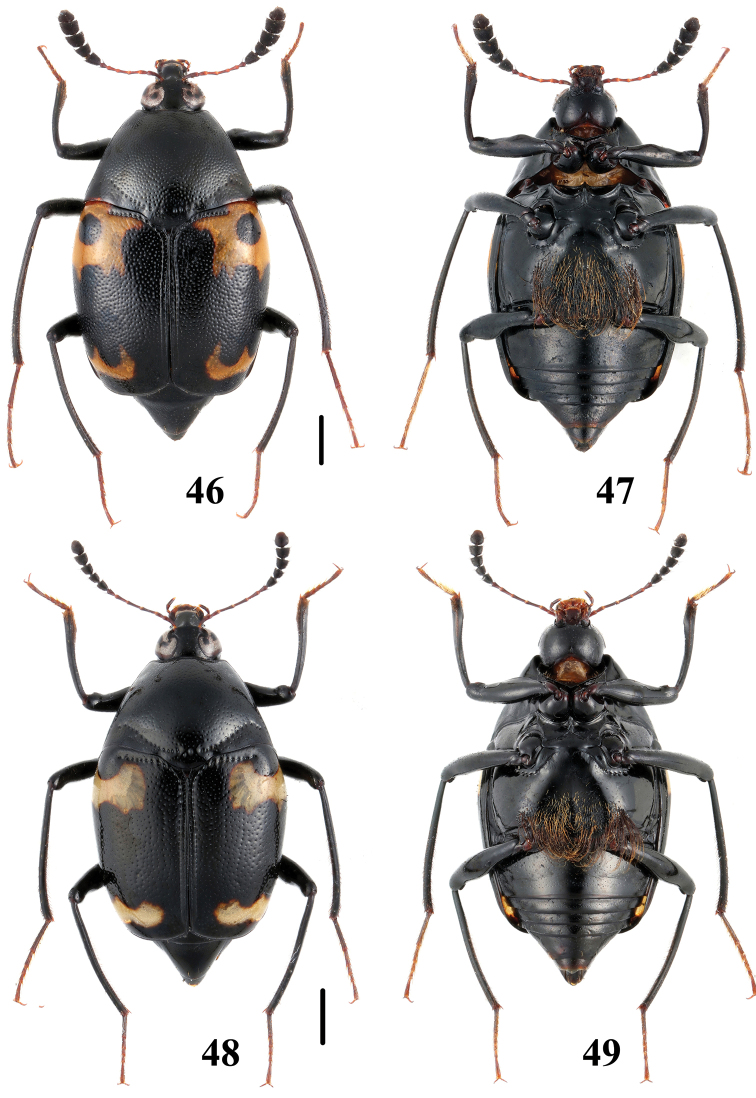
Habitus of *Scaphidium*. **46, 47** *Scaphidium stigmatinotum* **48, 49** *Scaphidium wuyongxiangi*. Scales = 1 mm.

Figs 46 , 47 , 122–125

Scaphidium stigmatinotum Löbl, 1999: 719; He et al. 2008b: 60; Tang and Li 2013: 179.

#### Material examined.

**Shaanxi:** 2♂♂6♀♀, Zhouzhi County, Qinling, Houzhenzi, 33°50'613"N, 107°50'183"E, alt. 1336m, 17–19.VII.2009, Huang & Xu leg. (SHNU); 2♂♂, Zhashui County, Yingpan Town, Hongmiaohe Vil., 33°82'189"N, 108°98'289"E, alt. 1110m, 3.VI.2007, H.-L. Shi leg. (IOZ). **Hunan:** 1♂, Mangshan, 25.IV.1977, J.-Y. Wu leg. (SEM) **Anhui:** 5♂♂8♀♀, Yuexi County, Yaoluoping, alt. 1050–1650m, 16–19.VI.2013, Dai & Peng leg. (SHNU) **Jiangsu:** 1♀, Nanjing City, Zijinshan, 10.VII.2005, Y. Huang leg. (SHNU); 1♀, Nanjing City, Zijinshan, 14.V.2006, L. Tang leg. (SHNU) **Zhejiang:** 1♀, Lin’an City, Tianmushan, alt. 300m, 10.VI.2007, Y.-X. Wu leg. (SHNU); 1♀, ibidem, 30°19'10"N, 119°26'51"E, alt. 410m, 21.X.2013, X.-B. Song leg. (SHNU); 1♀, Xianju County, Danzhu, alt. 450–600m, 2.VI.2006, Li & Shen leg. (SHNU); 1♀, Zhuji City, Caotazheng, Qiandashan, 29°39'04"N, 120°08'19"E, alt. 140m, under moss, 17.III.2012, T. X. Zhao leg. (CZTX); 1♂2♀♀, ibidem, from fungi, 30.IV.2012, T. X. Zhao leg. (CZTX); 1♂, ibidem, from fungi, 13.V.2012, T. X. Zhao leg. (CZTX); 1♀, Kaihua County, Gutianshan, 29°15'N, 118°8'E, 21.VI.2013, X.-B. Song leg. (SHNU) **Fujian:** 1♂, Jianning, Jinraoshan, 11.VI.1959, Jin & Lin leg. (SEM); 1♂1♀, Wuyishan City, Guadun, 24°44'2"N, 117°38'15"E, alt. 1200–1300m, 28.V.2012, X.-B. Song, leg. (SHNU) **Guangdong:** 1♀, Ruyuan County, Nanling N. R., alt. 1050m, 15.VII.2012, Ning & Yu leg. (SHNU); 1♀, ibidem, 1.VII.2009, L. Tang leg. (SHNU); 1♂, ibidem, 23.VIII.2009, L. Tang leg. (SHNU); 1♂, Shixing County, Chebaling, Xianrendong Vil., N24°73'478, E114°20'727", alt. 1508m, 26.VII.2008, H.-B. Liang leg. (IOZ); 1♂, Lian County, Nanling, Dadongshan, 3.VI.1998, Ouyang leg. (SYSU); 1♂, ibidem, 1.VIII.2007, H.-D. Chen leg. (SYSU); 1♂, ibidem, 26.VI.2009, R.-X. Jiang leg. (SYSU) **Guangxi:** 2♂♂1♀, Lingui County, Huaping N. R., Anjingping, alt. 1320m, 12.VII.2011, Peng & Zhu leg. (SHNU); 1♂1♀, Damingshan, 23°23'N, 108°29'E, alt. 1200–1300m, 30.VII.2012, J.-Y. Hu & X.-B. Song leg. (SHNU).

#### Distribution.

China (Shaanxi, Hunan, Anhui, Jiangsu, Zhejiang, Fujian, Guangdong, Guangxi, Yunnan).

#### Remarks.

This is new record to Anhui. The type locality: Yizu shan in original description is a typo error, which should be Jizushan of Central Yunnan. The species is characterized by the extremely dense punctation on the dorsum of the body and by one black dot entirely sealed in the basal fascia of the elytra.

### Scaphidium wuyongxiangi

He, Tang & Li, 2008

http://species-id.net/wiki/Scaphidium_wuyongxiangi

Figs 48 , 49 , 126–129 , 166

Scaphidium wuyongxiangi He, Tang & Li, 2008b: 57; Tang and Li 2013: 178.

#### Material examined.

**Holotype.** **Zhejiang:** ♂, Lin’an City, Tianmushan, 15~28.VIII.2003, Tang & Hu leg. (SHNU) **Paratypes. Zhejiang:** 1♂, Anji County, Longwangshan, 27.IV.2006, Tang & He leg. (SHNU); 1♀, Lin’an City, Tianmushan, alt. 300–400m, 11–15.VI.2007, HU Jia-Yao & WANG Yong-Yin leg. (SHNU); 1♀, ibidem, alt. 1000m, 19.V.2006, L. Ding leg. (SHNU); 2♂♂, Changhua Town, Qingliangfeng N. R., alt. 900m, 13.V.2008, BI Wen-Xuan leg. (SHNU) **Anhui:** 1♂7♀♀, Guniujiang, alt. 950–1050m, 28.IV.2005, Tang & Hu leg. (SHNU).

#### Other material.

**Anhui:** 1♂1♀, Yuexi County, Yaoluoping, alt. 1050–1650m, 17–19.VI.2013, Dai & Peng leg. (SHNU) **Zhejiang:** 1♂, Lin’an City, West Tianmushan, 5.VI.2008, X.-B. Song leg. (SHNU); 3♂♂1♀, ibidem, alt. 1000m, 7.VIII.2010, L. Tang leg. (SHNU); 1♀, ibidem, 12.V.2012, Li leg. (SHNU); 1♂, ibidem, 30°23'N, 119°25'E, alt. 1450m, 8.IX.2012 (SHNU); 2♂♂2♀♀, Lin’an City, East Tianmushan, alt. 1050–1150m, 13.IV.2011, Peng & Zhu leg. (SHNU); 1♂1♀, Anji County, Tonghanggang, 30°24'N, 119°26'E, alt. 1480m, 10.VI.2012, J.-Q. Zhu leg. (SHNU); 2♂♂1♀, Anji County, Longwangshan, Qianmutian, 30°24'N, 119°25'E, alt. 1050–1250m, 7.VI.2012, Hu, Yin & Ning leg. (SHNU); 3♂♂4♀♀, ibidem, 30°23'59"N, 119°26'26"E, alt. 1350m, 14.V.2013, Song & Tang leg. (SHNU); 1♀, Qingliangfeng, Longtangshan, Jiupu Vil., alt. 600–1000m, 19.VII.2009, Z.-W. Yin leg. (SHNU); 1♀, Longquan City, Fengyangshan, alt. 1500m, 10.VIII.2008, W.-X. Bi leg. (SHNU) **Fujian:** 3♂♂1♀, Wuyishan City, Guadun, N 27°45', E117°28', alt. 1800 m, 1.VI.2012, Peng & Dai leg. (SHNU) **Jiangxi:** 2♀♀, Yichun City, Mingyueshan, 27°35'44"N, 114°16'26"E, alt. 1140m, 23.X.2013, Peng, Shen & Yan leg. (SHNU); 4♂♂, Luxi County, Yangshimu, 27°33'58"N, 114°14'24"E, alt. 1230m, 25.X.2013, Peng, Sheng & Yan leg. (SHNU); 15♂♂9♀♀, Luxi County, Wugongshan, 27°27'55"N, 114°10'10"E, alt. 1280m, 28.X.2013, Peng, Shen & Yan leg. (SHNU) **Sichuan:** 1♂1♀, Mingshan County, Mendingshan, alt. 1400m, 6.VIII.2009, Tang & He leg. (SHNU).

#### Distribution.

China (Zhejiang, Anhui, Jiangxi, Fujian, Sichuan).

#### Remarks.

This is new record to Jiangxi. The species is characterized by its dumbbell-shaped fascia of elytra.

### Scaphidium vernicatum

(Pic, 1954)

http://species-id.net/wiki/Scaphidium_vernicatum

**Figures 50–53.**
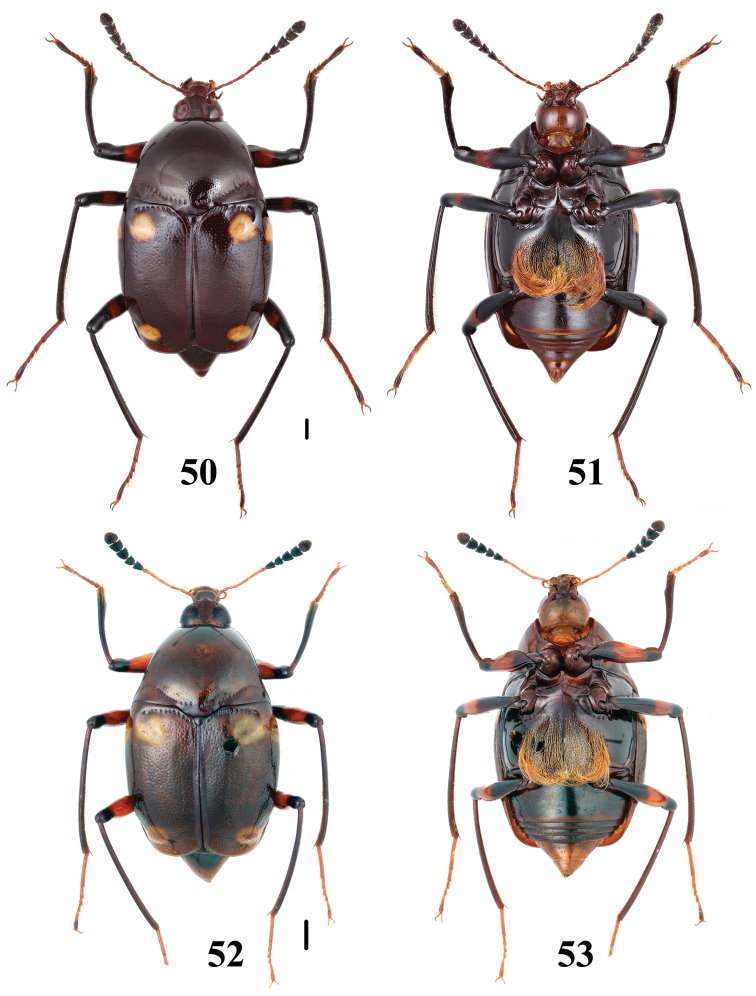
Habitus of *Scaphidium*. **50, 51** *Scaphidium vernicatum* **52, 53** *Scaphidium perpulchrum*. Scales = 1 mm.

Figs 50 , 51 , 130–133

Scaphium vernicatum Pic, 1954c: 57; Löbl 1999: 711.

#### Material examined.

**Paralectotype.** **Fujian:** 1♀, Kuatun, 4.V.1946. (MHNG).

#### Other material.

**Fujian:** 3♂♂2♀♀, Wuyishan City, Guadun, alt. 1100–1500m, 29.V.–1.VI. 2012, Song, Peng & Dai leg. (SHNU)

#### Distribution.

China (Fujian, Jiangxi).

#### Remarks.

The species is the largest species in China, with the BL 8.7–14.3 mm. It is extremely similar to *Scaphidium perpulchrum* Csiki, 1909 from Vietnam and no striking difference is known between them, though an immature male specimen of *Scaphidium perpulchrum* identified by Löbl has larger elytral fascia and faint coloration of body. The species is also similar to *Scaphidium direptum* Tang & Li, 2010 and *Scaphidium connexum* sp. n., but differs from them by the larger body size and bicolored legs, and from *Scaphidium connexum* also by the subhumeral fascia consisting of two separated yellow dots which are connected in *Scaphidium connexum*.

### Scaphidium direptum

Tang & Li, 2010

http://species-id.net/wiki/Scaphidium_direptum

**Figures 54–57.**
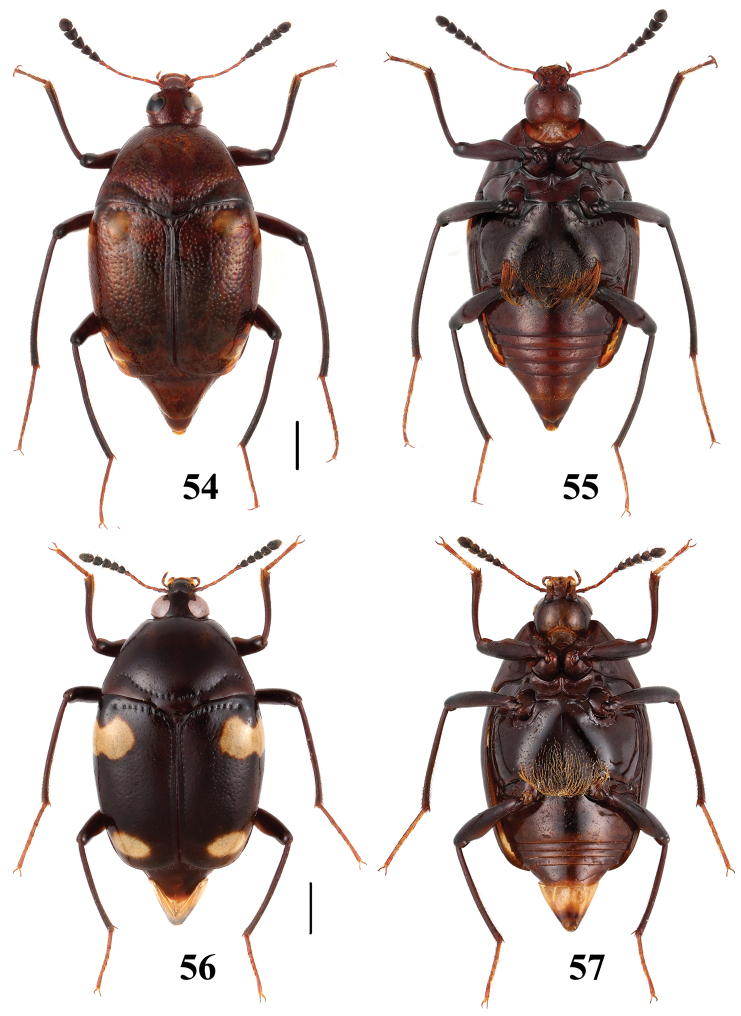
Habitus of *Scaphidium*. **54, 55** *Scaphidium direptum* **56, 57** *Scaphidium connexum*. Scales = 1 mm.

Figs 54 , 55 , 134–137

Scaphidium direptum Tang & Li, 2010b: 318.

#### Material examined.

**Holotype.** **Guangdong:** ♂, Shixing County, Chebaling N. R., alt. 365–500m, 23–26. VII.2008, X.-Y. Zhu leg. (SHNU).

#### Paratypes.

**Guangdong:** 1♂1♀, same data as for the holotype. (SHNU) **Fujian:** 1♀, Wuping County, Liangyeshan N. R., alt. 510m, 19.XI.2006, W.-J. He leg. (SHNU).

#### Other material.

**Guangdong:** 1♂, Leqing County, Bijiashan, alt. 120–200m, 16–18.X.2003, K.-B. Deng leg. (SYSU) **Guangxi:** 1♀, Shangsi County, Shiwandashan, alt. 300–500m, 25.IV.2011, Zhai, Peng & Zhu leg. (SHNU)

#### Distribution.

China (Guangdong, Fujian, Guangxi).

#### Remarks.

This is a new record for Guangxi. The species is similar to *Scaphidium vernicatum*, *Scaphidium perpulchrum* and *Scaphidium connexum*; for differences see remarks under *Scaphidium vernicatum*.

### Scaphidium connexum sp. n.

http://zoobank.org/C0043E72-CCA0-4FED-AF4F-71BED5D27885

http://species-id.net/wiki/Scaphidium_connexum

Figs 56 , 57 , 138–141

Scaphidium vernicatum : Tang & Li, 2010b: 320 (misidentification).

#### Type material.

**Holotype.** **Zhejiang:** ♂, Kaihua County, Gutianshan, 29°15'N, 118°8'E, alt. 800m, 21.VI.2013, X.-B. Song leg. (SHNU).

#### Paratypes.

**Zhejiang:** 3♂♂1♀, same data as for the holotype (1 pair in MHNG, remaining in SHNU); 1♂, ibidem, 29°14'N, 118°8'E, alt. 400–500m, 19.VI.2013, X.-B. Song leg. (SHNU). **Fujian:** 1♀, Fuzhou City, Beifeng, 4.III.2004, M. Li leg. (SHNU); 1♂, Wuyishan, Guadun, alt. 1200m, 9.VI.2009, Y. Huang leg. (SHNU); 3♀♀, ibidem, N27°44', E117°38", alt. 1300–1500m, 27–29.V.2012, Peng & Dai leg. (SHNU). **Guangxi:** 1♂, Shangsi County, Shiwandashan, alt. 300–500m, 23.IV.2011, Zhai, Peng & Zhu leg. (SHNU); 1♂, Xing’an County, Maoershan, 25°48'N, 110°24'E, alt. 450m, 25.VII.2012, J.-Y. Hu leg. (SHNU).

#### Description.

BL: 5.4–7.1 mm.

Body dark brown with antennal segments I–VI and tarsi lighter. Antennal club (Fig. 140) blackish with terminal segment slightly lighter in apical 1/3. Each elytron with two yellowish fasciae: dumbbell-shaped subhumeral fascia and round subapical fascia.

Frons coarsely and densely punctate, punctures on vertex coarser and denser than those on remaining surface, puncture intervals of vertex mostly smaller than half puncture diameter.

Pronotum slightly raised above elytra. Antebasal puncture row impressed, more or less interrupted at middle, with punctures coarse and regular; discal punctation coarser than that of vertex, puncture intervals as broad as 0.5 to 1.5 puncture diameters.

Elytra with disc slightly impressed apically, basal and sutural stria rows impressed, without discal puncture row; discal punctation similar to that of pronotum except on slightly denser punctuated apical impression; basal stria row with punctures slightly coarser than those forming pronotal antebasal row, sutural stria puncture row very fine.

Prohypomera moderately uneven, with relatively coarse, sparse and shallow punctures.

Mesepisterna sparsely and distinctly punctate.

Abdominal tergites and sternites with relatively coarse and dense punctures. and dense microsculpture consisting of micropunctures.

Legs moderately long, mesotibiae and metatibiae moderately curved.

Male. Metaventrite (Fig. 57) impressed at middle, with long and suberect pubescence. Profemur (Fig. 141) with ventral side roundly expanded in basal 1/5 to basal 3/5, forming two ridges. Protibia (Fig. 141) gradually wider starting from basal 1/3, without distinct protuberance at widest point, slightly narrowed toward apex. Median lobe of aedeagus (Fig. 138) with sclerotized internal sac (Fig. 139) consisting of apical and basal complex of sclerites.

#### Distribution.

China (Zhejiang, Fujian, Guangxi).

#### Remarks.

The new species was once misidentified as *Scaphidium vernicatum* in Tang and Li 2010b. It is characterized by its elytra with the subhumeral fascia consisting of two connected yellow dots.

#### Etymology.

The Latin adjective “connexum” refers to its subhumeral fascia of elytra consisting of two connected yellow dots.

### Key to *Scaphidium* species of East China

**Table:** 

1	Body entirely black, sometimes with bluish metallic tint, without fascia	2
–	Body coloration different, pronotum and/or elyta with fasciae or uniformly reddish	6
2	Larger species, BL≥5.9 mm; meso- and metafemora black with reddish fasciae	3
–	Smaller species, BL≤5.1 mm; meso- and metafemora blackish or reddish without fascia	4
3	Larger species, BL: 7.3–9.7 mm; male profemora and protibiae (Fig. 73) without angle. Habitus (Figs 9, 10), characters (Figs 70–73). China (Chongqing, Sichuan, Guizhou, Hunan, Zhejiang, Fujian, Guangdong, Yunnan, Guangxi, Hainan, Taiwan?), Nepal, Myanmar, Thailand, Laos, Malaysia, Vietnam, Indonesia	*Scaphidium grande*
–	Smaller species, BL: 5.9–7.3 mm; male profemora (Fig. 77) with an acute angle at apical third, protibiae (Fig. 77) with a blunt angle before apical angle. Habitus (Figs 11, 12), aedeagus (Figs 74–77). China (Anhui)	*Scaphidium spinatum*
4	Body form elongate with lateral sides somewhat parallel; elytra evenly punctate without puncture row.	5
–	Body form oval; elytra with a puncture row consisting of coarse punctures. BL: 4.1–4.5 mm. Habitus (Figs 7, 8), characters (Figs 66–69). China (Fujian)	*Scaphidium fukiense*
5	Body black without metallic luster; male metaventrite with setiferous patch. BL: 3.5–4.4 mm. Habitus (Figs 5, 6), characters (Figs 62–65). China (Zhejiang, Hunan, Hubei, Guangxi, Hainan), North Korea	*Scaphidium comes*
–	Body black with strong bluish metallic luster; male metaventrite without setiferous patch. BL: 4.1–4.9 mm. Habitus (Figs 1, 2), characters (Figs 58–61). China (Zhejiang, Anhui, Chongqing)	*Scaphidium jinmingi*
6	Elytra reddish-brown and without fascia, pronotum usually with black dots. BL: 3.7–4.6 mm. Habitus (Figs 18, 19), characters (Figs 17, 82–85). China (Zhejiang, Anhui)	*Scaphidium varifasciatum*
–	Elytra with fasciae, pronotum with or without fasciae	7
7	Pronotum reddish-yellow with pair of black fasciae or at least blackish at base	8
–	Pronotum blackish or brownish without fascia	14
8	Elytra each with a humeral black dot	9
–	Elytra without humeral black dot. BL: 3.4–4.3 mm. Habitus (Figs 20, 21), characters (Figs 86–89). China (Zhejiang, Anhui, Fujian, Jiangxi, Guangdong, Guangxi)	*Scaphidium sauteri*
9	Elytra each with a inner basal black dot	10
–	Elytra without inner basal black dot	11
10	Body larger, BL: 5.7–6.4 mm; terminal antennal segment entirely yellowish. Habitus (Figs 40, 41), characters (Figs 110–113). China (Fujian, Chongqing, Guizhou, Guangxi, Yunnan)	*Scaphidium robustum*
–	Body smaller, BL: 4.6–5.7 mm; terminal antennal segment yellowish in apical third. Habitus (Figs 37–39), characters (Figs 106–109). China (Zhejiang, Anhui, Hunan, Hubei, Jiangxi, Guizhou, Sichuan, Yunnan, Guangxi)	*Scaphidium biwenxuani*
11	Pronotum reddish-yellow with basal portion blackish, pronotal fasciae usually appeared. BL: 4.1–5.9 mm. Habitus (Figs 29–33), characters (Figs 98–101). China (Zhejiang, Fujian, Jiangxi, Hunan, Guangxi)	*Scaphidium sinense*
–	Pronotum entirely reddish-yellow with pair of pronotal fasciae	12
12	Elytra each with a black humeral dot and a large oval black mark on median portion. BL: 4.4–5.1 mm. Habitus (Figs 13–15), characters (Figs 16, 82–85). China (Zhejiang, Fujian, Jiangxi, Guangxi)	*Scaphidium crypticum*
–	Elytra each with 4 or 5 black dots	13
13	Elytra each with 2 long puncture rows. BL: 5.1–6.1 mm. Habitus (Figs 22–25), characters (Figs 90–93). China (Jiangxi, Fujian, Guangdong, Yunnan, Guangxi, Hainan, Taiwan)	*Scaphidium formosanum*
–	Elytra each with 2 long puncture rows and 2 short rows between long puncture rows and sutural puncture row. BL: 4.2–5.9 mm. Habitus (Figs 26–28), characters (Figs 94–97). China (Hubei, Fujian, Sichuan, Guangxi, Yunnan, Hainan), Myanma.	*Scaphidium carinense*
14	Larger species, BL≥5.4 mm; pronotal punctation dense and deep	15
–	Smaller species, BL: 4.8–5.8 mm; pronotal punctation sparse and shallow. Habitus (Figs 34–36), characters (Figs 102–105). China (Zhejiang, Hunan, Hubei, Anhui, Sichuan, Yunnan, Guangxi, Guangdong)	*Scaphidium delatouchei*
15	Elytra with one or two black dots entirely sealed in basal fascia	16
–	Elytra without black dot sealed in subhumeral fascia	18
16	Elytra each with 1 black dot sealed in basal fascia. BL: 6.0–8.2 mm. Habitus (Figs 46, 47), characters (Figs 122–125). China (Shaanxi, Hunan, Anhui, Jiangsu, Zhejiang, Fujian, Guangdong, Guangxi, Yunnan)	*Scaphidium stigmatinotum*
–	Elytra each with 2 black dots sealed in basal fascia	17
17	Inner black dot near scutellum reaching basal stria. BL: 6.8–8.6 mm. Habitus (Figs 44, 45), characters (Figs 118–121). China (Zhejiang, Fujian)	*Scaphidium klapperichi*
–	Inner black dot near scutellum departing from basal stria. BL: 6.8–7.5 mm. Habitus (Figs 42, 43), characters (Figs 114–117). China (Anhui)	*Scaphidium bayibini*
18	Elytra with subapical fascia transverse and tridentate anteriorly. BL: 5.9–8.0 mm. Habitus (Figs 48, 49), characters (Figs 126–129). China (Zhejiang, Anhui, Jiangxi, Fujian, Sichuan)	*Scaphidium wuyongxiangi*
–	Elytra with subapical fascia round	19
19	Elytra with subhumeral fascia consisting of two connected yellow dots. BL: 5.4–7.1 mm. Habitus (Figs 56, 57), characters (Figs 138–141). China (Zhejiang, Fujian, Guangxi)	*Scaphidium connexum*
–	Elytra with subhumeral fascia consisting of two separate yellow dots	20
20	Body blackish-brown, each femur black with narrow reddish fascia. BL: 8.7–14.3 mm. Habitus (Figs 50–51), characters (Figs 130–133). China (Fujian, Jiangxi)	*Scaphidium vernicatum*
–	Body reddish-brown, femora entirely dark reddish-brown. BL: 6.9–7.8 mm. Habitus (Figs 54, 55), characters (Figs 134–137). China (Guangdong, Fujian, Guangxi)	*Scaphidium direptum*

**Figures 58–65.**
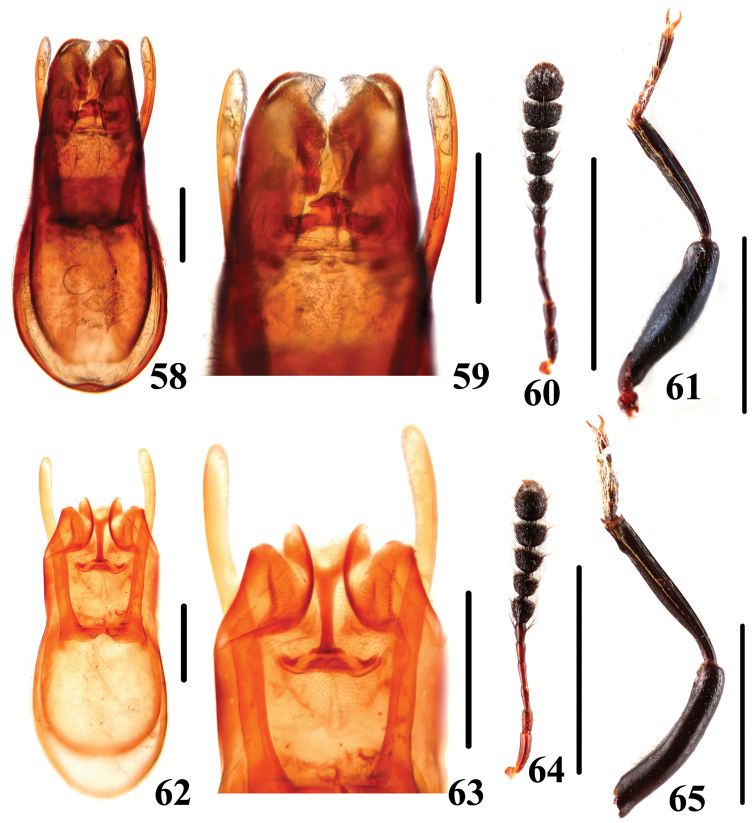
Characters of *Scaphidium*. **58–61** *Scaphidium jinmingi* **62–65** *Scaphidium comes* **58, 59, 62, 63** aedeagus **60,64** antenna **61, 65** male foreleg. Scales = 0.25 mm (**58, 59, 62, 63**); scales = 1 mm (**60, 61, 64, 65**).

**Figures 66–73.**
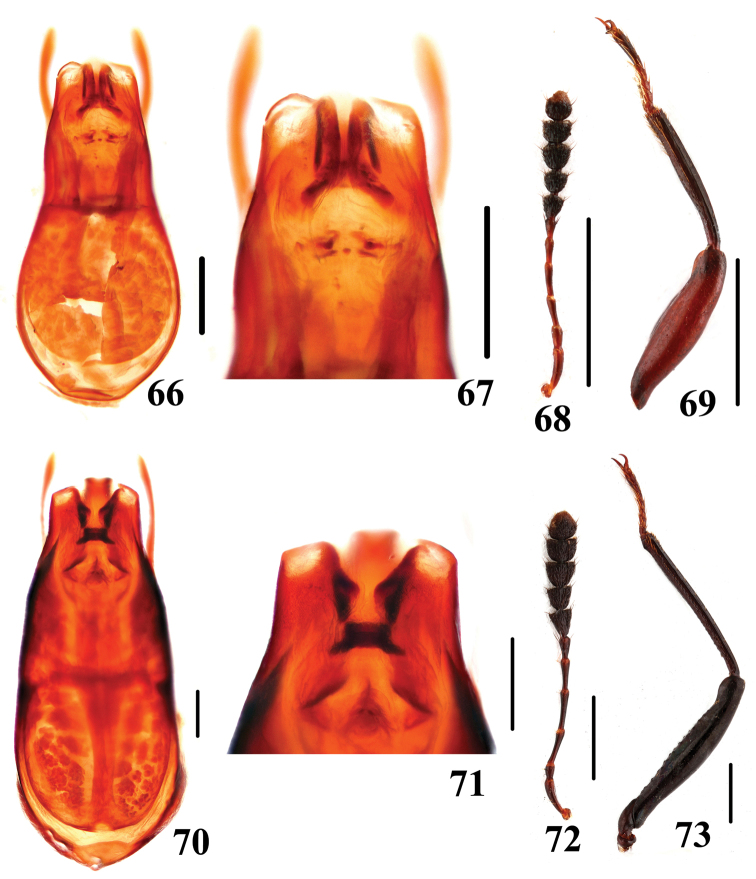
Characters of *Scaphidium*. **66–69** *Scaphidium fukiense* **70–73** *Scaphidium grande* **66, 67, 70, 71** aedeagus **68, 72** antenna **69, 73** male foreleg. Scales = 0.25 mm (**66, 67, 70, 71**); scales = 1 mm (**68, 69, 72, 73**).

**Figures 74–81.**
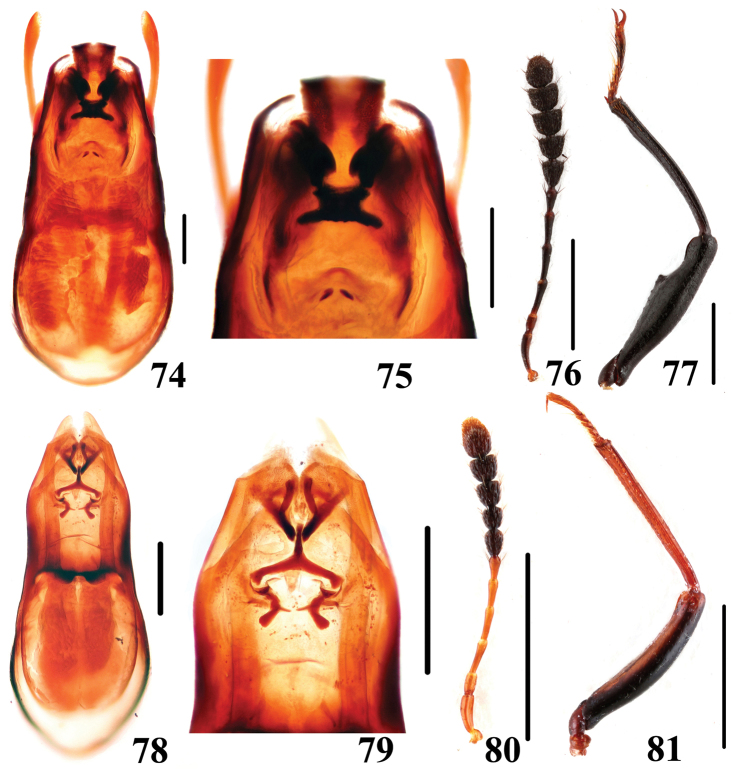
Characters of *Scaphidium*. **74–77** *Scaphidium spinatum* **78–81** *Scaphidium crypticum* **74, 75, 78, 79** aedeagus **76, 80** antenna **77, 81** male foreleg. Scales = 0.25 mm (**74, 75, 78, 79**); scales = 1 mm (**76, 77, 80, 81**).

**Figures 82–89.**
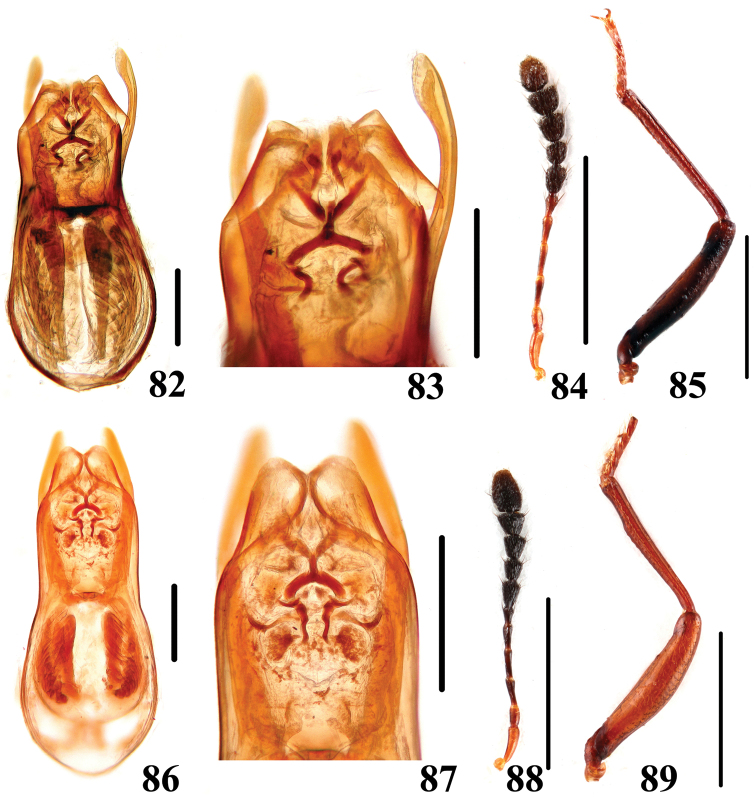
Characters of *Scaphidium*. **82–85** *Scaphidium varifasciatum* **86–89** *Scaphidium sauteri* **82, 83, 86, 87** aedeagus **84, 88** antenna **85, 89** male foreleg. Scales = 0.25 mm (**82, 83, 86, 87**); scales = 1 mm (**84, 85, 88, 89**).

**Figures 90–97.**
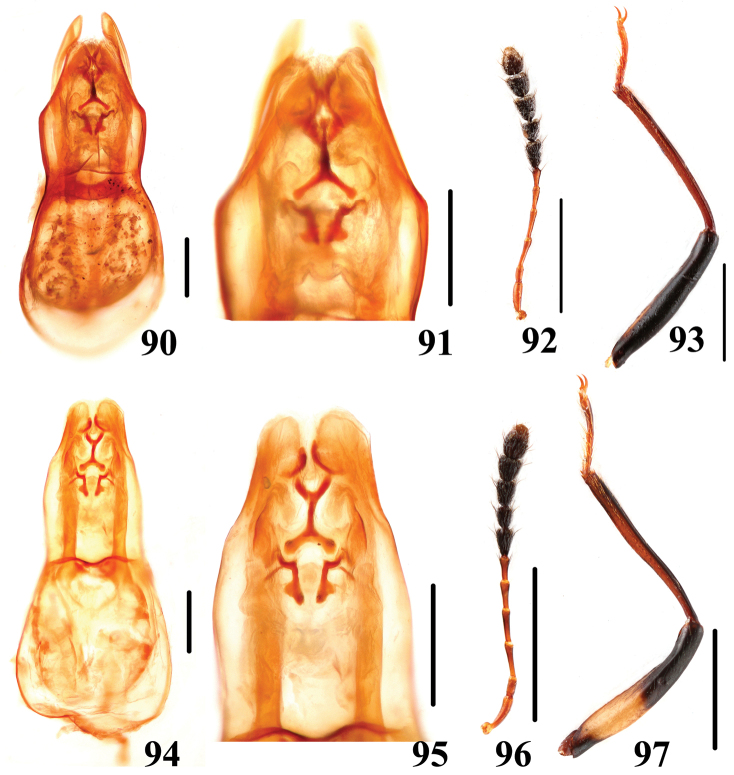
Characters of *Scaphidium*. **90–93** *Scaphidium formosanum* **94–97** *Scaphidium carinese* **90, 91, 94, 95** aedeagus **92, 96** antenna **93, 97** male foreleg. Scales = 0.25 mm (**90, 91, 94, 95**); scales = 1 mm (**92, 93, 96, 97**).

**Figures 98–105.**
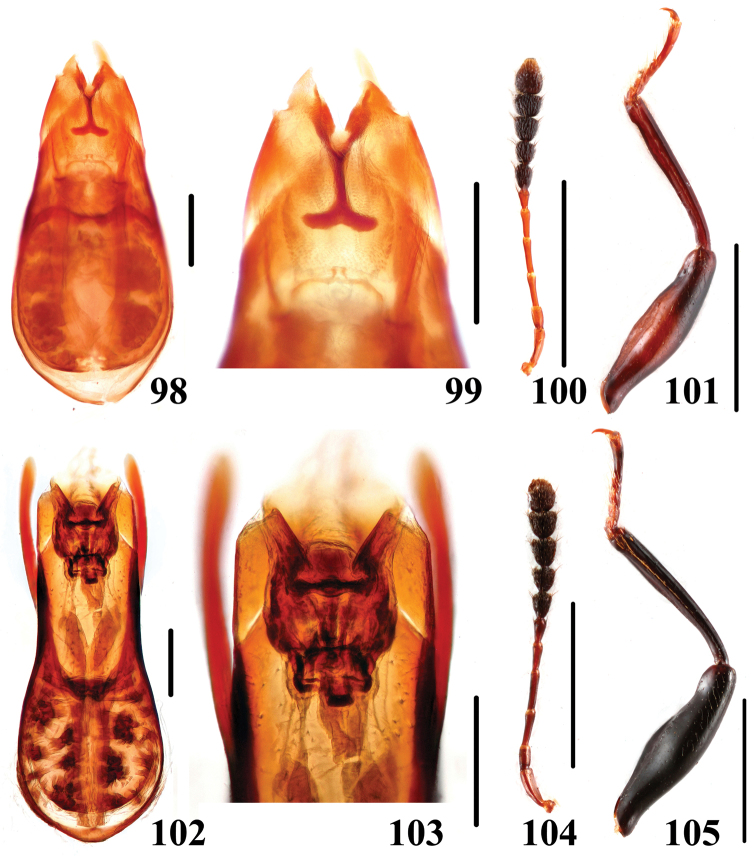
Characters of *Scaphidium*. **98–101** *Scaphidium sinense* **102–105** *Scaphidium delatouchei* **98, 99, 102, 103** aedeagus **100, 104** antenna **101, 105** male foreleg. Scales = 0.25 mm (**98, 99, 102, 103**); scales = 1 mm (**100, 101, 104, 105**).

**Figures 106–113.**
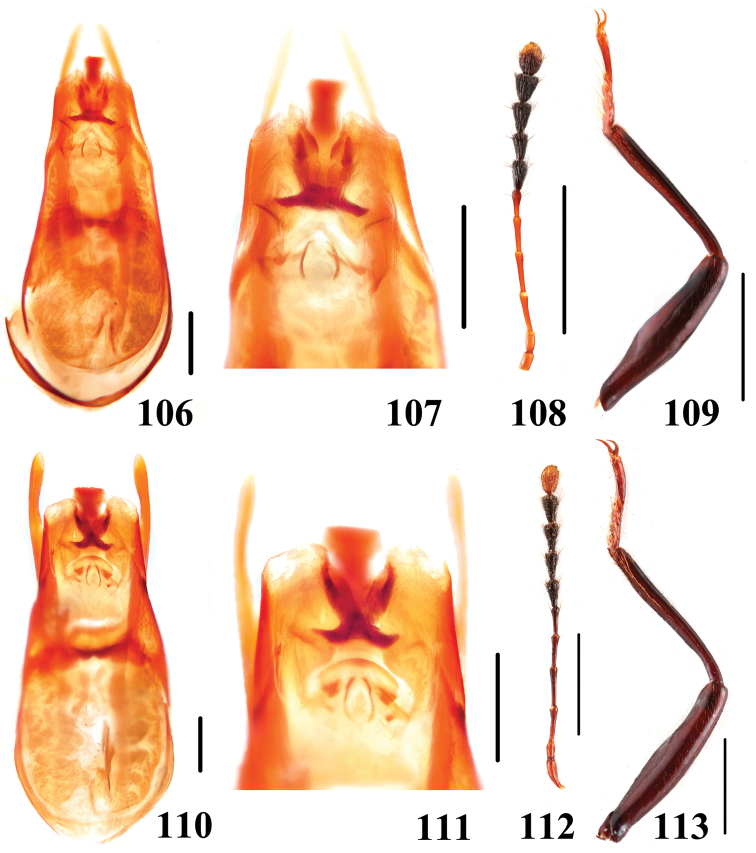
Characters of *Scaphidium*. **106–109** *Scaphidium biwenxuani* **110–113** *Scaphidium robustum* **106, 107, 110, 111** aedeagus **108, 112** antenna **109, 113** male foreleg. Scales = 0.25 mm (**106, 107, 110, 111**); scales = 1 mm (**108, 109, 112, 113**).

**Figures 114–121.**
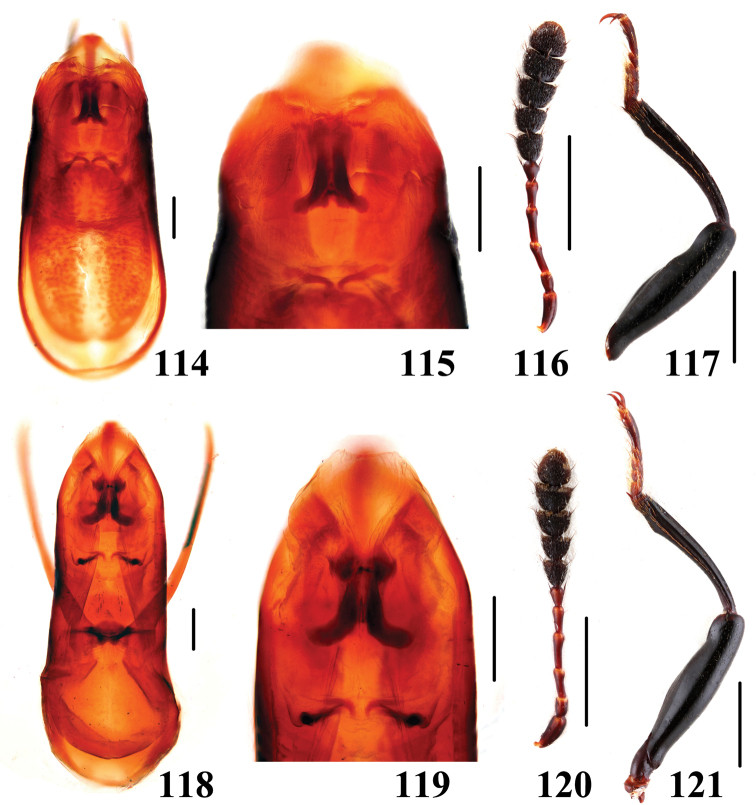
Characters of *Scaphidium*. **114–117** *Scaphidium bayibini* **118–121** *Scaphidium klapperichi* **114, 115, 118, 119** aedeagus **116, 120** antenna **117, 121** male foreleg. Scales = 0.25 mm (**114, 115, 118, 119**); scales = 1 mm (**116, 117, 120, 121**).

**Figures 122–129.**
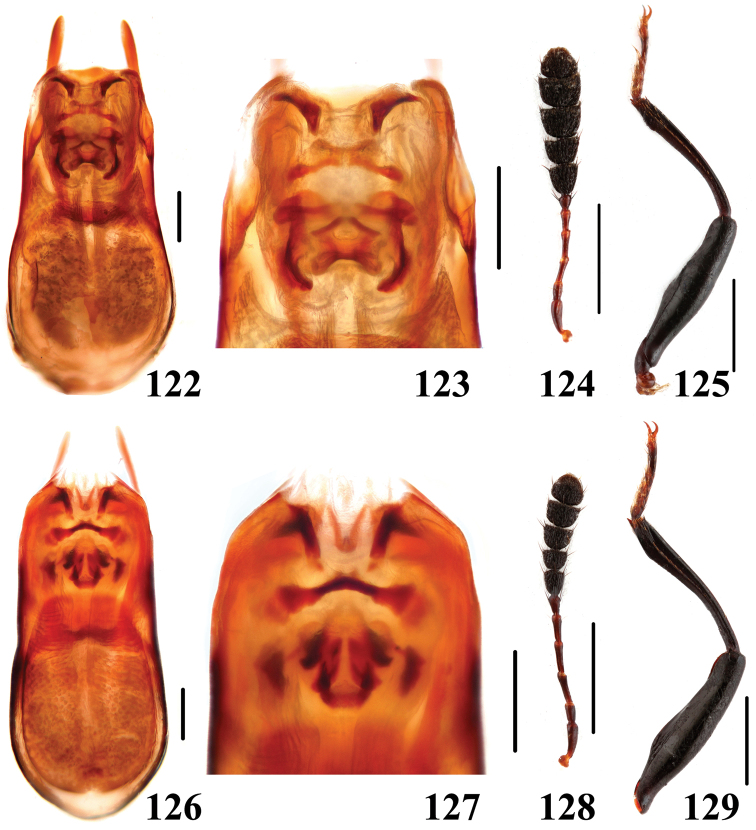
Characters of *Scaphidium*. **122–125** *Scaphidium stigmatinotum* **126–129** *Scaphidium wuyongxiangi* **122, 123, 126, 127** aedeagus **124, 128** antenna **125, 129** male foreleg. Scales = 0.25 mm (**122, 123, 126, 127**); scales = 1 mm (**124, 125, 128, 129**).

**Figures 130–137.**
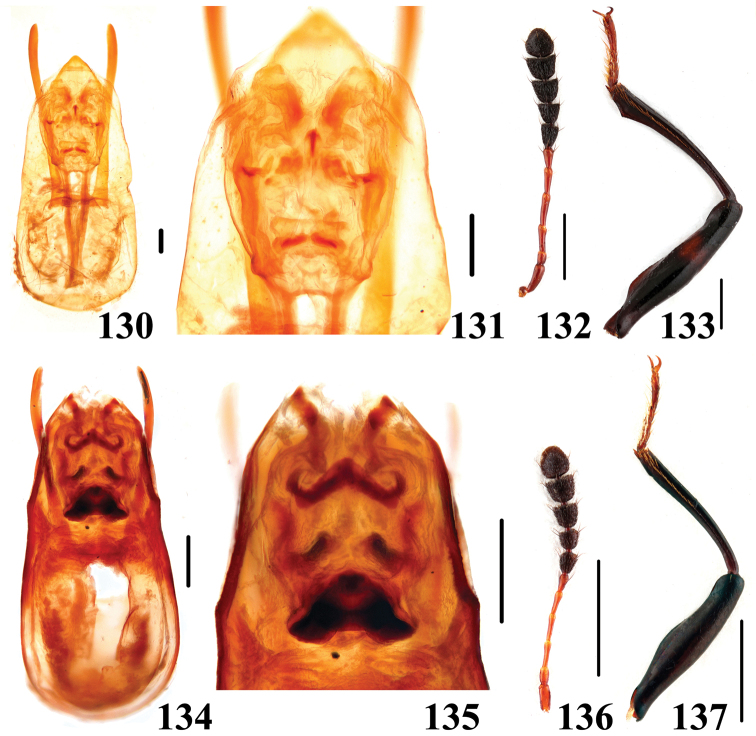
Characters of *Scaphidium*. **130–133** *Scaphidium vernicatum* **134–137** *Scaphidium direptum* **130, 131, 134, 135** aedeagus **132, 136** antenna **133, 137** male foreleg. Scales = 0.25 mm (**130, 131, 134, 135**); scales = 1 mm (**132, 133, 136, 137**).

**Figures 138–141.**
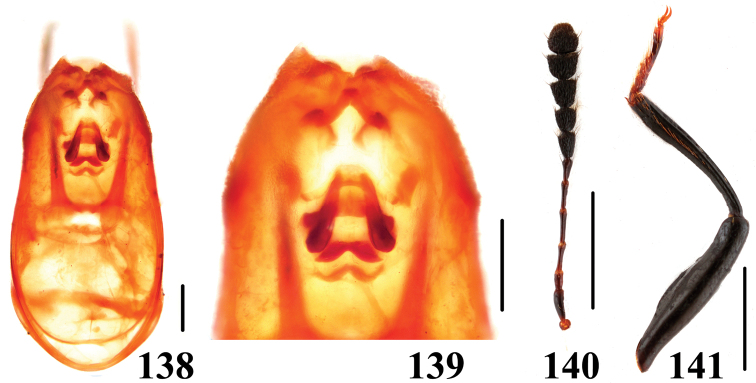
Characters of *Scaphidium connexum*. **138, 139** aedeagus **140** antenna **141** male foreleg. Scales = 0.25 mm (**138, 139**); scales = 1 mm (**140, 141**).

**Figures 142–155.**
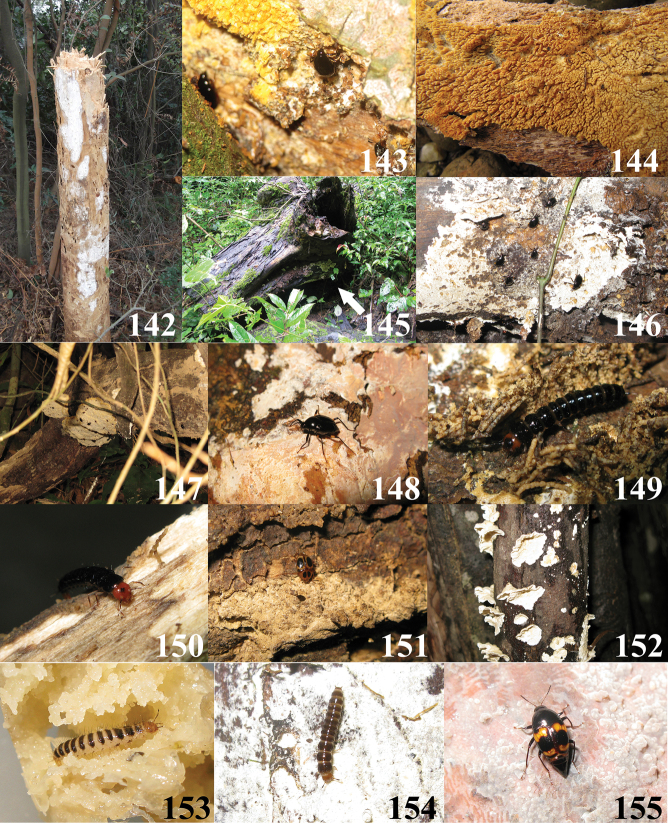
**142** Habitat and host fungi of *Scaphidium comes* (Photo by Mr. Zhong Peng from Hunnan, Xiangtan City, Zhaoshan County at 30.I.2011) **143,144** Host fungi and *Scaphidium comes* (Photo by Mr. Jian-Qing Zhu from Guangxi, Shangsi County, Shiwandashan at 23.IV.2011) **145, 146** Host fungi and *Scaphidium grande* (Photo by Mr. Jia-Yao Hu from Guangxi, Dayaoshan at 22.VII.2011) **147** Habitat and host fungi of *Scaphidium grande* (Photo by Mr. Jian-Qing Zhu from Guangxi, Shangsi County, Shiwandashan at 23.IV.2011) **148** Host fungi and *Scaphidium grande* (Photo by Mr. Liang Tang from Guangxi, Xing’an County, Mao’ershan at 7.VII.2011) **149, 150** Larvae of *Scaphidium grande* (Photo by Mr. Liang Tang from Guangxi, Xing’an County, Mao’ershan at 8.VII.2011) **151** host fungi of *Scaphidium crypticum* (Photo by Mr. Liang Tang from Guangxi, Shangsi County, Shiwandashan at 4.V.2011); **152** host fungi of *Scaphidium crypticum* (Photo by Mr. Zi-Wei Yin from Zhejiang, Qingyuan County, Baishanzu at 8.VII.2009) **153** Larva of *Scaphidium carinense* (Photo by Mr. Liang Tang in lab at 18.VII.2011, larva collected from Guangxi, Xing’an County, Mao’ershan) **154** Larva of *Scaphidium sinense* (Photo by Mr. Liang Tang from Zhejiang, Lin’an City, West Tianmushan at 10.VIII.2010) **155** Host fungi of *Scaphidium delatouchei* (Photo by Mr. Liang Tang from Zhejiang, Lin’an City, West Tianmushan at 4.VIII.2010).

**Figures 156–166.**
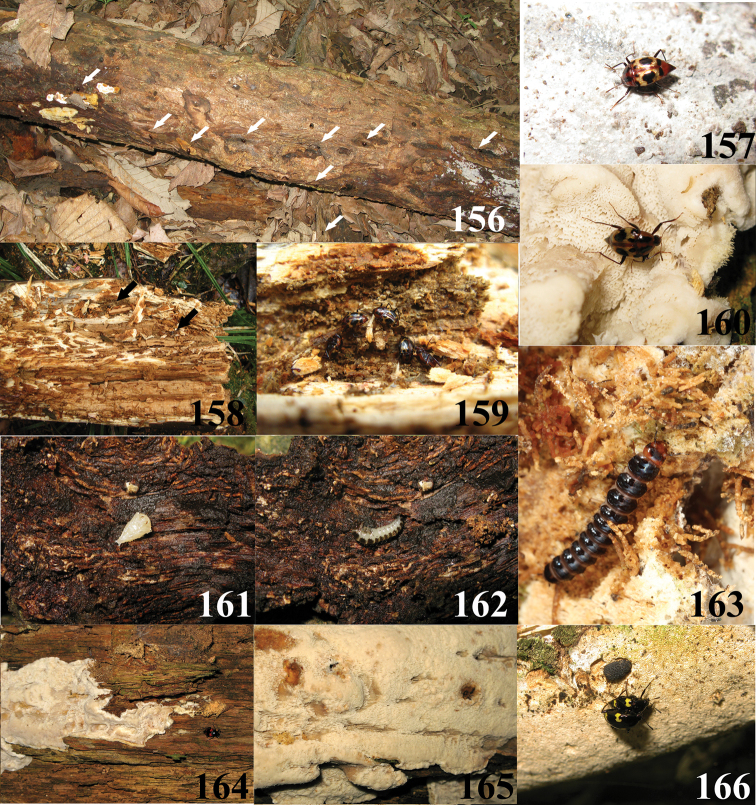
**156** Habitat of *Scaphidium biwenxuani* (Photo by Mr. Liang Tang from Zhejiang, Anji County, Longwangshan at 14.V.2013) **157** host fungi of *Scaphidium biwenxuani* (Photo by Mr. Liang Tang from Zhejiang, Lin’an City, West Tianmushan at 22.VIII.2009) **158, 159** overwinter population of *Scaphidium biwenxuani* in cerambycid galleries (Photo by Mr. Wen-Xuan Bi from Zhejiang, Lin’an City, West Tianmushan at 29.I.2009) **160** host fungi of *Scaphidium biwenxuani* (Photo by Mr. Liang Tang from Zhejiang, Qingyuan County, Baishanzu at 23.IX.2008) **161, 162** Prepupa and pupa of *Scaphidium biwenxuani* (Photo by Mr. Liang Tang from Zhejiang, Lin’an City, West Tianmushan at 9.VIII.2010); **163** larva of *Scaphidium spinatum* (Photo by Mr. Liang Tang in lab at 2.VII.2013, larva collected from Zhejiang, Yuexi County, Yaoluoping) **164, 165** host fungi of *Scaphidium klapperichi* (Photo by Mr. Liang Tang from Zhejiang, Qingyuan County, Baishanzu at 23.IX.2008) **166** host fungi and *Scaphidium wuyongxiangi* (Photo by Mr. Liang Tang from Zhejiang, Anji County, Longwangshan at 14.V.2013)

## Biological notes

The most abundant species of *Scaphidium* in East China are *Scaphidium biwenxuani* (Fig. 156) and *Scaphidium wuyongxiangi*, both also distributed in western and southern parts of China, but apparently as smaller populations. *Scaphidium grande* is probably the most widely distributed species in Asia, thriving in only South China and present in middle-latitude zones as small scattered populations. Almost 20 specimens of *Scaphidium grande* from Mao’ershan (Guangxi) collected from a large pile of logs along a road far from lush forests indicates its strong dispersal ability. *Scaphidium stigmatinotum* is the most widely distributed species in China, but always found in small populations. This species was even collected in some depauperate secondary forests at low altitude and near cities, e.g. Nanjing and Zhuji, where none of other species has been found, and this may imply its strong endurance of adverse environments.

Most species of *Scaphidium* from East China live in mountain areas, above 800m altitudes, especially during mid and late summer (July and October) which may be hot (sometimes reaching 38 °C) and dry in the lowlands. Only few specimens of *Scaphidium comes*, *Scaphidium delatouchei*, *Scaphidium sinense* and *Scaphidium wuyongxiangi* were recorded during this period from the lowlands (about 300m altitudes), while many more species occur in the highlands (about at 1000m altitudes), and in relatively larger numbers. There are also few records of *Scaphidium comes*, *Scaphidium klapperichi*, *Scaphidium sinense* and *Scaphidium stigmatinotum* from the lowlands and found in spring or autumn. In several attempts at rearing *Scaphidium biwenxuani* and *Scaphidium sinense*, larvae were successfully matured at 28–33 °C, showing that at least some species can support such temperatures. Obviously, habitat preferences are determined by the presence of fungi, which depend on temperature and moisture.

Both adults and larvae of *Scaphidium* feed on polyporaceous fungi (Figs 142–166) and usually can be found by searching logs with fungi; two specimens of *Scaphidium delatouchei* (Fig. 155) were collected from possibly different, undetermined fungi. The fungi and habitat preferences of *Scaphidium jinmingi* and *Scaphidium comes* seem to be somewhat different from other species, as most of these two species were gathered on fungus under bark.

Adults of*Scaphidium* are lucifugous. During the daytime the adults are mostly observed on shaded fungi. Few specimens of *Scaphidium wuyongxiangi* and *Scaphidium jinmingi* were collected by sweeping which indicates the adults may fly and repose on vegetation during the day. In very dense forests or after nightfall, they are active and reckless. *Scaphidium delatouchei* was observed slowly flying and hovering at dusk. Many adults may be found during night walking on exposed fungi. At that time, they are usually very calm, even under the flashlights and collectors are invisible to them. None was found to be attracted by lamp traps. Larvae are also sensitive to lights. They hide during the day in fecal retreats (see Leschen 1994), and exit to feed during the night.

The reaction of adults towards threats is different between the species, and depends also on temperature. Generally, adults tend to catalepsy and drop into leaf litter below at lower temperatures while they rather more frequently fly away at higher temperature. The length of catalepsy also depends on temperature. Usually it lasts briefly, but can be much longer at lower temperature. Some species are more alert than others. *Scaphidium grande* can notice a collector from meters away and may rapidly fly away, like flies (Figs 145, 146). It is very common to find *Scaphidium sinense* and *Scaphidium biwenxuani* on the same log in Tianmushan, and individuals of *Scaphidium sinense* are always more active and fly away first while *Scaphidium biwenxuani* prefers to hide on the opposite side of the log or in cavities. Disturbed adults of *Scaphidium* usually fly for few meters only to rest on surrounding vegetation. They usually fly back to the host fungi after few minutes, if not further disturbed.

The occurrence of larvae is irregular in East China and they may be found consistently from late spring to earlier autumn. Prepupa and pupa of *Scaphidium biwenxuani* (Figs 161, 162) were found under loose bark during summer. Several species were successfully reared from larvae to adults. Their photos (Figs 149, 150, 153, 154, 163) are provided in this paper as preliminary information. The adults overwinter usually hidden under bark. Several individuals of *Scaphidium wuyongxiangi* from Longwangshan (Zhejiang) were collected under bark in spring when they were still in hibernation. Many specimens of *Scaphidium comes* from Hunan were collected in January on fungi under the bark of a coniferous tree trunk (Fig. 142). *Scaphidium biwenxuani* was found in January, gathering in galleries of Cerambycidae (Figs 158, 159).

## Supplementary Material

XML Treatment for Scaphidium jinmingi

XML Treatment for Scaphidium comes

XML Treatment for Scaphidium fukiense

XML Treatment for Scaphidium grande

XML Treatment for Scaphidium spinatum

XML Treatment for Scaphidium crypticum

XML Treatment for Scaphidium varifasciatum

XML Treatment for Scaphidium sauteri

XML Treatment for Scaphidium formosanum

XML Treatment for Scaphidium carinense

XML Treatment for Scaphidium sinense

XML Treatment for Scaphidium delatouchei

XML Treatment for Scaphidium biwenxuani

XML Treatment for Scaphidium robustum

XML Treatment for Scaphidium bayibini

XML Treatment for Scaphidium klapperichi

XML Treatment for Scaphidium stigmatinotum

XML Treatment for Scaphidium wuyongxiangi

XML Treatment for Scaphidium vernicatum

XML Treatment for Scaphidium direptum

XML Treatment for Scaphidium connexum
